# A naturalistic, non-invasive method for capturing biometric data during autism evaluations

**DOI:** 10.3389/fpsyt.2026.1819384

**Published:** 2026-06-11

**Authors:** Khaleel Kamal, Janka Hatvani, Máté Pethő, András Sárkány, Imola Hamvas, Camille Brune, Allison L. Wainer, Emily Dillon, Elizabeth Berry Kravis, Edith Vanessa Ocampo, Zachary Enos Arnold, Iman Ghazal, Fatema Al-Faraj, Máté Csákvári, Kristóf Katona-Pucsek, Péter Kun, Dóra Oláh, Ferenc Hernáth, Attila Schulc, Anikó Mezősi, Alejandro Latorre, Fouad Al-Shaban, Latha Valluripalli Soorya, Zoltán Tősér

**Affiliations:** 1 Argus Cognitive, Inc., Hanover, NH, United States; 2 Department of Psychiatry and Behavioral Sciences, Rush University Medical Center, Chicago, IL, United States; 3 Carroll University, Waukesha, WI, United States; 4 Departments of Pediatrics, Neurological Sciences, Anatomy and Cell Biology, Rush University Medical Center, Chicago, IL, United States; 5 Treatment and Education of Autistic and Related Communication Handicapped Children (TEACCH) Autism Program, Department of Psychiatry, The University of North Carolina at Chapel Hill, Chapel Hill, NC, United States; 6 Neurological Disorders Research Center, Qatar Biomedical Research Institute (QBRI), Hamad Bin Khalifa University (HBKU), Qatar Foundation, Doha, Qatar; 7 HBKU Core Labs, Hamad Bin Khalifa University (HBKU), Doha, Qatar

**Keywords:** ASD, autism spectrum disorder, behavioral analysis, biometric data, diagnostic support, machine learning, pediatrics

## Abstract

**Introduction:**

This study evaluated a machine learning tool designed to non-intrusively quantify and analyze biometric data of gaze, facial expressions, and paralinguistic social communication features during standardized autism observational assessments. The primary aim was to assess the diagnostic accuracy of this multimodal tool in capturing key social communication features of autism in a diverse neurodevelopmental disabilities cohort and neurotypical (NT) cohort, ages 2-12.

**Methods:**

The study enrolled 546 participants across four sites in the USA (n=246) and Qatar (n=300). Of these, 458 (83.6%) met quality indicators for both video and audio recordings and comprised the analysis set. Primary outcome measures were diagnostic accuracy, sensitivity and specificity relative to reference diagnoses. Random Forest classifiers were trained using a developmentally-adaptive approach: separate models for three developmental groups (few-to-no words, phrase speech, fluent speech) using 97 biometric features from video, audio and gaze data. Performance was assessed via leave-one-out cross-validation on the inner set (n=338) and validated on an independent hold-out test set (n=120).

**Results:**

Classification between ASD (idiopathic and syndromic) and non-ASD (clinical and NT) participants achieved 77.8% sensitivity and specificity in the inner set. When distinguishing ASD from NT participants alone, the sensitivity and specificity both increased to 82.0%. In the hold-out test set, the model demonstrated 62.3% sensitivity and 81.4% specificity for ASD versus non-ASD, and 72.1% sensitivity and 88.6% specificity for ASD versus NT. Performance varied by demographics in the case of sex, with males showing higher sensitivity (79% to 75%) and females showing higher specificity (84% to 70%).

**Discussion:**

This study demonstrates the feasibility of using semi-automated multimodal computational analysis to quantify multi-modal autism social communication behaviors and distinguish ASD from NT in clinically and ethnically diverse samples. Known difficulties with differential diagnosis in non-ASD neurodevelopmental conditions with autism-like features remain a limitation requiring further development. Data suggest promise for such tools to support task-sharing models within existing clinical approaches.

## 1 Introduction

Early diagnosis of autism spectrum disorder (ASD) enables timely intervention and support, and the potential to yield enhanced outcomes, for both individuals and their families (1–3). Despite advances in understanding the biological mechanisms contributing to autism risk, diagnosis remains primarily behavioral, based on careful differentiation of clinical characteristics. The gold standard for clinical diagnosis, the Autism Diagnostic Interview-Revised (ADI-R) (4) and structured behavioral observations using the Autism Diagnostic Observation Schedule-Second Edition (ADOS-2) (5, 6) demonstrate compelling capacity to distinguish autism from non-autism clinical populations and provide invaluable data to inform phenotyping approaches in research and clinical care (7).

However, these approaches were designed for specialty care settings over 20 years ago when autism prevalence was approximately 1 in 125 and have not been scaled to meet current needs. Autism prevalence has reached 1 in 31 children in the United States in 2022 (8) and roughly 1 in 100 children worldwide (9). While children are eventually diagnosed, autism assessment pathways in the United States are often resource-intensive and associated with substantial delays between initial concern, screening, and formal diagnosis, reflecting persistent limitations in specialist service capacity (10). This problem is even more pronounced in low- and middle-income countries (11).

The complex and diverse nature of ASD limits easy translation to non-specialist settings, underscored by a 39% false-negative rate when clinicians relied solely on reviewing video clips without standardized coding (12). In turn, standardized scoring systems based on highly trained rater observations remain subject to limitations in resources, clinical expertise, and cultural context (13). These diagnostic challenges are compounded by the heterogeneity of autism and the presence of overlapping clinical features (core and associated) in related neurodevelopmental conditions (14, 15). Prior studies have shown that experienced clinicians maintain relatively high diagnostic confidence across demographic factors such as sex and socio-demographic background, whereas uncertainty increases in children with mid-level verbal abilities and moderate autism symptom severity, where presentations are diagnostically less distinct (16).

Today, when autism is the second most common childhood mental health condition, task-sharing models provide opportunities to increase accessibility of autism diagnostic tools in non-specialist settings (e.g., primary care, educational settings) (17–19). Computational approaches and machine learning tools for autism detection have demonstrated diagnostic utility and promise to address current demands. However, many existing models remain limited to controlled settings that restrict movement or capture isolated behavioral channels, such as eye gaze alone or single-angle video coding, while also having less cultural diversity, as experiments were conducted only on a single populace (e.g. US populace) (18, 20–22). To address these limitations, a machine learning model was designed to non-intrusively quantify and analyze biometric data of social, language, and motor behaviors during autism evaluations. Recent work on multimodal behavioral analysis during ADOS-2 assessments has demonstrated the feasibility of combining vision-, speech-, and gaze-based features to support the detection of autism-related behavioral markers in semi-structured clinical interactions, highlighting the potential of automated systems to extract clinically relevant cues from naturalistic diagnostic settings (23).

This paper outlines the validation of the system which extracts a subset of social-communicative behaviors observed during ADOS-2 sessions from audio and video data. The primary goal was to evaluate the clinical relevance and predictive accuracy of these features by assessing their capability to support ASD classification when trained against a reference standard diagnosis. The long-term goal of this effort is to create a framework applicable to video data collected under various fixed observational protocols in non-specialist settings.

## 2 Materials and methods

### 2.1 Study design

Three groups of children, ages 2-12, were recruited for this study: children with autism spectrum disorder (ASD), children with non-autism clinical conditions including neurodevelopmental and psychiatric conditions (non-ASD clinical), and children without neurodevelopmental or psychiatric presentations (neurotypical, NT). Participants were recruited across two countries and four sites: Rush University Medical Center and Sago Clinical Research in Chicago, Illinois, U.S., and Qatar Biomedical Research Institute and the Shafallah Center in Doha, Qatar. Written informed consent was obtained from parents or legal guardians of all participants. Institutional review boards approved study protocols at Rush University Medical Center (Approval No. 19031905-IRB01) and Qatar Biomedical Research Institute (Approval No. QBRI-IRB 2021-07-096).

### 2.2 Evaluation procedure

Demographic and medical history data were collected from all participants. Screening measures included the Social Responsiveness Scale-2 (24) at US sites and the Social Communication Questionnaire (SCQ) (25) at Qatar sites. US participants completed age-appropriate cognitive testing (26) (WISC, 2014; WASI-II, 1999; or DAS-Early Years, 2018) and adaptive behavior assessment (Vineland-3). At the Qatar sites, cognitive and adaptive behavior assessments were administered only when clinically indicated. Additional cognitive/adaptive characterization was supported by the Vineland-3 Adaptive Behavior Composite at both sites, with scores below 70 flagged as having potential intellectual and developmental disability (ID/DD) (**Supplementary Tables 1**, **2**).

All participants received the ADOS-2 (Modules T, 1, 2, 3) in English or Arabic, administered by research reliable raters. To further ensure consistency across sites, inter-site reliability was established between the US and Qatar sites prior to and during data collection in Qatar. Based on the methodologies described, expert, doctoral-level clinicians established reference diagnoses based on DSM-5 criteria, integrating direct behavioral assessments, developmental questionnaires, and parent interviews. It should be noted that a comprehensive, standardized medical history form was not available for all participants in Qatar. Clinical information was primarily limited to documentation of a previous ASD diagnosis and reported comorbid conditions. Where possible, families provided official medical records. Reference clinical diagnoses served as the final classification when data from the ADOS-2, clinical diagnoses and/or screening instruments were discordant.

To assess the diagnostic utility of social communication features captured in the system (27), participants (n=546) were stratified into three groups: 1) ASD (n=265), 2) non-ASD (n=281), and 3) NT cohorts (n=200), a subset of non-ASD (**Supplementary Table 1**). ASD participants met DSM-5 criteria based on all available information, with potential co-morbidities. The non-ASD cohort included individuals with attention-deficit/hyperactivity disorder (ADHD), genetic syndromes (e.g., fragile X syndrome - FXS), other medical or physical conditions, and intellectual disability/developmental delay (ID/DD) and the NT participants. The NT cohort contained only NT participants.

### 2.3 Participants

A total of 546 participants were enrolled from June 2017 to March 2025 across the US (n = 246) and Qatar (n = 300).

Participants were eligible for inclusion if they had a chronological and/or mental age between 12 and 155 months. Individuals in the ASD group were required to have a documented clinical diagnosis of autism spectrum disorder or a professional recommendation for ASD evaluation, meet the DSM-5 diagnostic criteria, and have a positive reference diagnosis.

Participants in the non-ASD, clinical cohort were required to have a documented clinical diagnosis of a non-ASD clinical condition. At the Rush University site, this included participants with a confirmed genetic diagnosis of fragile X syndrome (FXS), defined as a full mutation (>200 CGG repeats in FMR1) based on DNA testing. Other non-ASD clinical participants met standardized measure-based inclusion criteria appropriate to their diagnosis (e.g., Connors Rating Scale for ADHD).

NT participants were required to have no history of medical, genetic, developmental, or psychiatric diagnoses.

Clinical exclusion criteria for all groups included: history of extreme prematurity (<1000 grams birth weight) with associated early neo-natal complications (e.g. intra-cerebral hemorrhage, prolonged hypoxia, prolonged hypoglycemia), a history of significant brain injury requiring hospitalization, seizure onset, or physical or sensory disabilities (e.g. significant vision or hearing loss) that limited the participation in the assessment. For the non-ASD clinical cohort recruited in Qatar, participants were additionally excluded if they had a comorbid ASD diagnosis.

Technical and procedural exclusion criteria included corrupted or missing audio or video data. Minimum data quality requirements included the availability of Tobii eye-tracking recordings and audio data, with at least 1 minute of usable audio containing ≥10% total voice activity and at least 3 minutes of detectable facial data in the video recordings. Additional exclusions applied if the ADOS-2 assessment was not administered, if the ADOS-2 session duration was less than 28 minutes, or if the Tobii recording duration was shorter than 50% of the corresponding stationary camera recording. Finally, participants were excluded if insufficient vocalization remained after two rounds of manual speaker diarization annotation.

**Figure 1:**
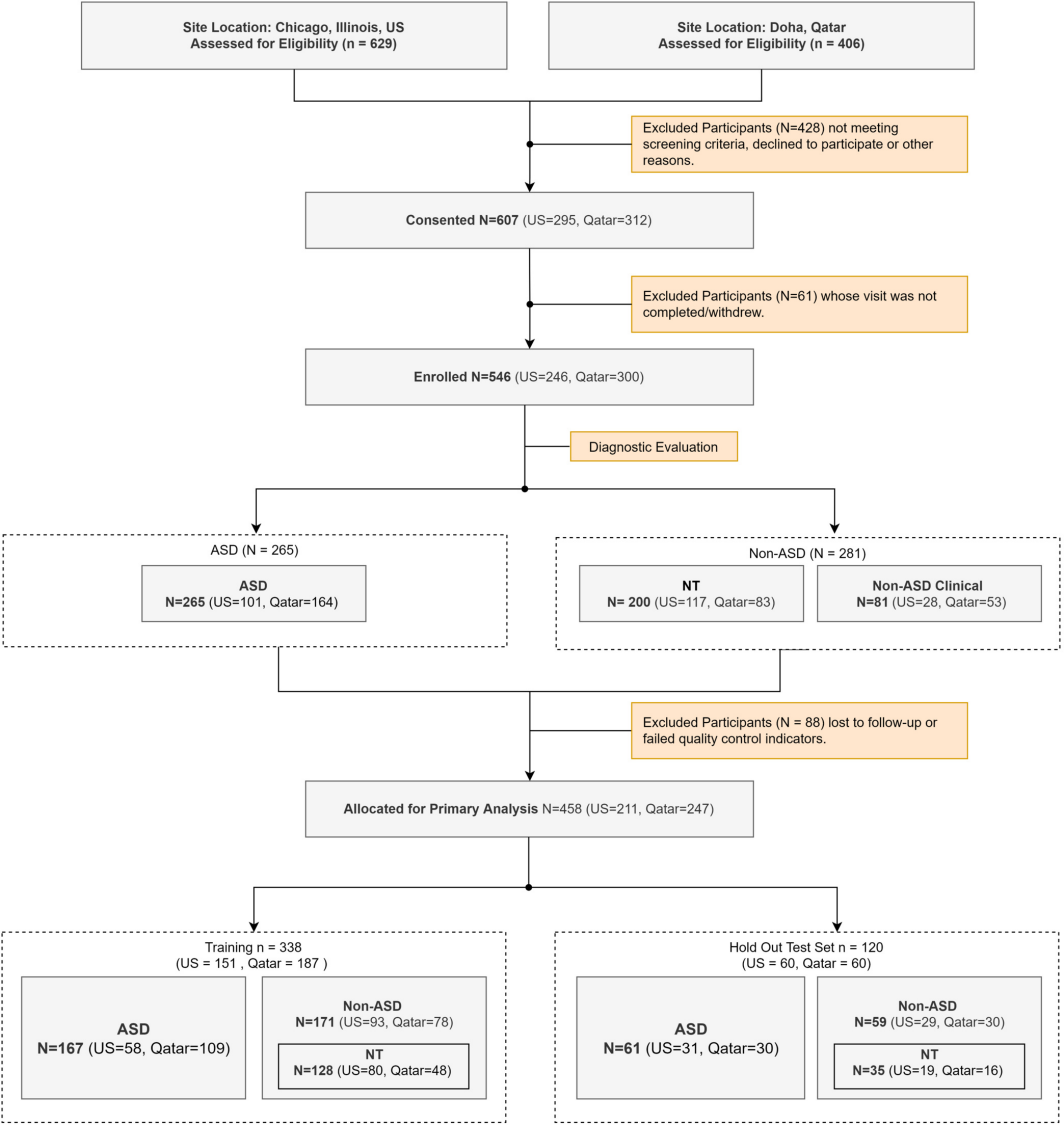
Flow diagram of participant recruitment and inclusion. Enrolled participants underwent expert clinical evaluation using standardized diagnostic assessments as well as multimodal biometric data collection via audio and video analysis. Further information on the inclusion and exclusion criteria can be found in the Methods.

Demographics and recruitment flow are presented in **Table 1**; **Figure 1**; **Supplementary Table 4**. Research data collected was coded with a participant’s unique identifier, and personally identifying data were removed. As the video data cannot be fully anonymized, all video recordings were stored on secure internal servers with restricted access.

**Table 1:** Participant characterization and demographics.

Characterization categories	Metric/subgroup	All participants (N = 546)			Analyzed participants (N = 458)		
		ASD	Non-ASD		ASD	Non-ASD	
				NT subset of non-autism			NT subset of non-autism
Count		265.0	281	200	228	230	163
Age, mo	Mean (SD)	86.83 (42.48)	71.69 (38.13)	70.99 (39.1)	85.26 (42.91)	71.32 (36.99)	69.22 (36.8)
	Median	88.0	64.0	66.0	85.5	65.5	65.0
Sex, No. (%)	Female	57 (21.51)	145 (51.6)	121 (60.5)	47 (20.61)	119 (51.74)	98 (60.12)
	Male	208 (78.49)	136 (48.4)	79 (39.5)	181 (79.39)	111 (48.26)	65 (39.88)
Race, No. (%) *	American Indian or Alaska Native	2 (0.75)	0 (0)	0 (0)	2 (0.88)	0 (0)	0 (0)
	Asian	5 (1.89)	1 (0.36)	1 (0.5)	3 (1.32)	0 (0)	0 (0)
	Black/African American	15 (5.66)	30 (10.68)	28 (14)	13 (5.7)	22 (9.57)	20 (12.27)
	White	31 (11.7)	87 (30.96)	73 (36.5)	27 (11.84)	74 (32.17)	65 (39.88)
	More than one race	4 (1.51)	4 (1.42)	3 (1.5)	4 (1.75)	4 (1.74)	3 (1.84)
	Unknown or not reported	208 (78.49)	159 (56.58)	95 (47.5)	179 (78.51)	130 (56.52)	75 (46.01)
Ethnicity, No. (%) **	Hispanic	21 (7.92)	33 (11.74)	29 (14.5)	19 (8.33)	25 (10.87)	25 (15.34)
	Non-Hispanic	39 (14.72)	87 (30.96)	74 (37)	32 (14.04)	73 (31.74)	61 (37.42)
	Unknown or not reported	205 (77.36)	161 (57.3)	97 (48.5)	177 (77.63)	132 (57.39)	77 (47.24)
ADOS-2 Social affect score	Mean (SD)	12.74 (4.75)	2.77 (3.24)	2 (2.63)	12.58 (4.74)	2.75 (3.24)	1.99 (2.71)
	Median	12.0	2.0	1.0	12.0	1.0	1.0
ADOS-2 RRB score	Mean (SD)	3.66 (2.12)	0.79 (1.16)	0.59 (0.99)	3.68 (2.16)	0.73 (1.09)	0.57 (0.96)
	Median	4.0	0.0	0.0	4.0	0.0	0.0
ADOS-2 Total score	Mean (SD)	16.28 (5.9)	3.49 (3.76)	2.54 (3.08)	16.26 (5.89)	3.48 (3.75)	2.56 (3.17)
	Median	16.0	2.0	1.5	16.0	2.0	2.0
ADOS-2 Comparison score	Mean (SD)	6.73 (1.87)	1.94 (1.53)	1.52 (1.11)	6.7 (1.9)	1.9 (1.48)	1.51 (1.09)
	Median	7.0	1.0	1.0	7.0	1.0	1.0
Vineland Adaptive Behavior Scale ***	Adaptive behavior, mean (SD)	63.8 (18.43)	95.2 (20.11)	105.97 (10.63)	63.31 (16.32)	95.19 (20.45)	105.88 (10.88)
Country of data collection	US	101	145	117	89	122	99
	Qatar	164	136	83	139	108	64

The Autism Diagnostic Observation Schedule, Second Edition (ADOS-2) includes the Social Affect and Restricted and Repetitive Behavior (RRB) domain scores. Non-ASD contains all non-ASD clinical and NT participants, while NT only contains NT participants. * Race metadata was only available for Rush samples. ** Ethnicity metadata was only available for Rush samples. ***Both Vineland-II and Vineland-3 were administered over the course of recruitment at Rush; data represent average Vineland scores across versions.

### 2.4 Group matching

Matching procedures differed by site. At the US site, FXS participants were frequency-matched to the ASD sample on chronological age across four strata (18–36, 37–60, 61–155, and 156–216 months), with the older stratum included to capture FXS participants whose mental age fell within the study’s developmental range despite exceeding the chronological age ceiling. NT participants were frequency-matched to the ASD sample using two parallel recruitment targets: one mirroring the ASD sample’s chronological age distribution across three strata (18–36, 37–60, and 61–155 months) and one mirroring the ASD sample’s mental age distribution across four strata (12–17, 18–36, 37–60, and 61–155 months), with an additional 12–17 month stratum included to accommodate younger MA-matched participants. At the Qatar site, NT participants were individually matched to ASD participants within a six-month chronological age window; non-ASD clinical participants were similarly matched when available.

### 2.5 Biometric extraction

**Figure 2:**
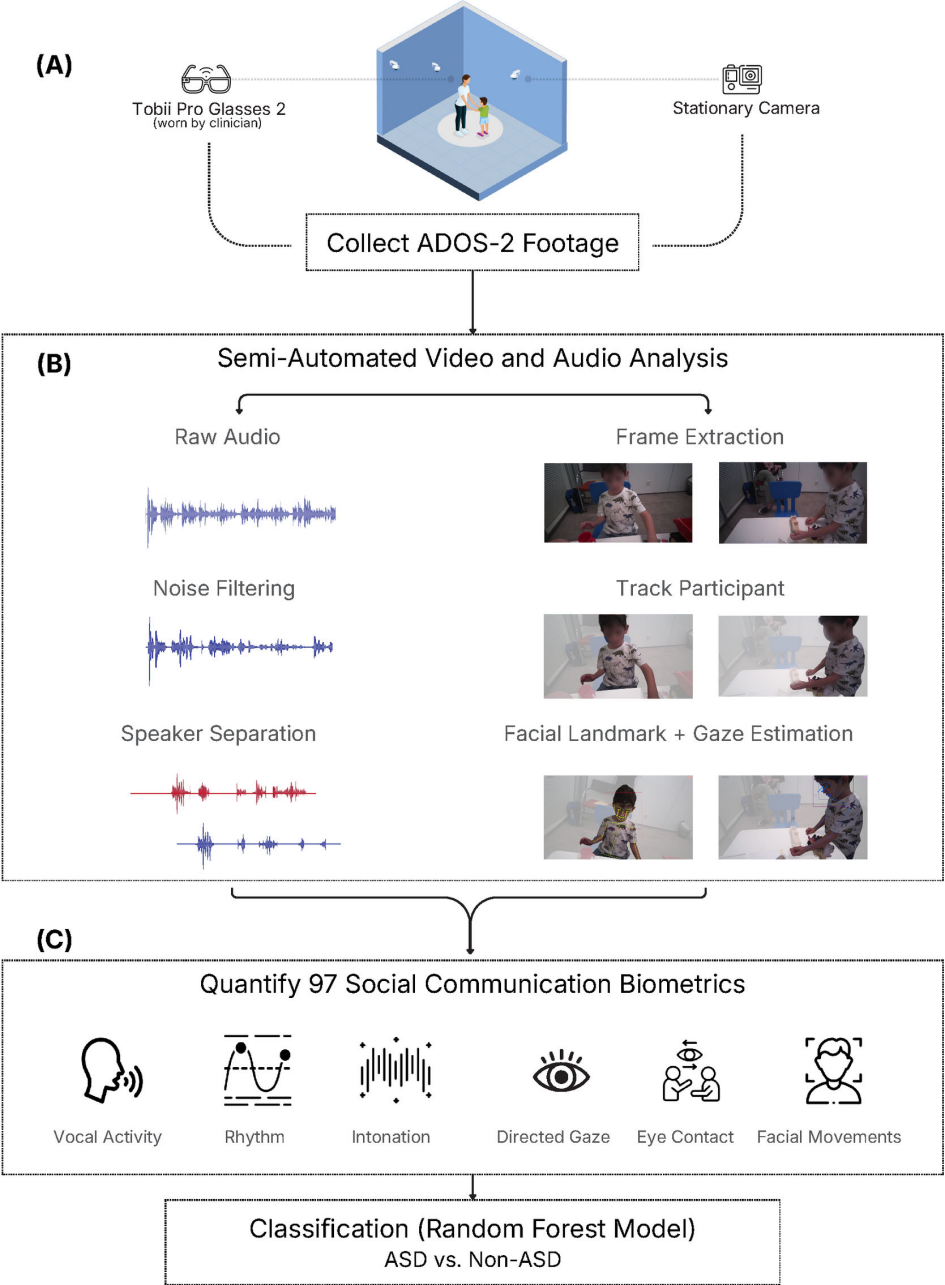
Overview of the multimodal data collection and biometric analysis pipeline. **(A)** ADOS-2 sessions were recorded using a stationary camera for audio capture and clinician-worn Tobii Pro Glasses 2 for point-of-view video and gaze data. **(B)** Semi-automated analysis processed audio (noise filtering, speaker separation) and video (frame extraction, participant tracking, facial landmark and gaze estimation) streams in parallel. Subject identification was the only step requiring human confirmation. **(C)** Ninety-seven social communication biometric features across six domains were computed and served as inputs to a Random Forest classifier for ASD versus non-ASD classification.

To quantify biometric correlates of social-communicative behavior during ADOS-2 assessments, a multi-modal feature extraction pipeline was implemented integrating video, audio, and eye-tracking data. Biometric extraction overlaid and was independent of ADOS-2 administration and scoring, with the assessment serving as a rich, standardized observational context for the data-capture. Data collection utilized stationary cameras for audio recording and a clinician-worn Tobii Glasses 2 eye-tracking device for video and gaze data. The Tobii Glasses tracked the gaze of the clinician, while participant gaze was tracked algorithmically using the video recordings from the glasses. A standardized camera calibration protocol ensured recording consistency across assessment sessions and sites. The Tobii glasses were calibrated during each session by the clinician by focusing on the center of the calibration target included in the Tobii kit. All video and audio data underwent automated analysis (**Figure 2**; **Supplementary Figures 1**–**4**), where only the participant identification step required human confirmation. For Module T, 1, and 2 participants, caregivers could be present during the session recordings, however, the participant identification step ensured that only the test participants’ data were retained for analysis. To establish our feature extraction framework, we cataloged behavioral markers routinely assessed by clinicians during ADOS-2 administration, identifying which criteria could be quantified through automated video and audio analysis, and defining the complete biometric feature set implemented in the analysis pipeline (**Table 2**).

**Table 2:** Excerpt of biometric features.

Characteristic	Metric group	Number of metrics	Biometric description
Social communication - paralinguistic metrics	IMF Minimum Intensity	19	The Intrinsic Mode Function (IMF) represents rhythmic components of speech, capturing fine-grained timing variations within the speech signal that contribute to overall speech rhythm.
	Envelope Spectrum (ENV)	10	The Envelope Spectrum (ENV) represents the amplitude envelope of speech over time, capturing temporal patterns in speech rhythm.
	Temporal Modulation Spectrum (TMS)	8	The Temporal Modulation Spectrum (TMS) measures the rate and pattern of rhythmic changes in speech over time, capturing nuanced characteristics related to speech rhythm and timing.
	Intonation	10	Intonation reflects the pitch contour of speech over time, capturing variations in fundamental frequency that convey prosodic patterns such as emphasis, question versus statement distinction, and emotional tone.
Social communication – vocalization	Mean Length Of an Utterance	4	Mean Length of Utterance (MLU) measures the average utterance length, indicating language complexity. Lower MLU may suggest simpler or delayed speech, while higher MLU reflects more advanced language use.
	Vocalization Duration Variability	1	Variability in the duration of vocalizations indicates the regularity or inconsistency of speech patterns. High variability could suggest erratic speech, which is often seen in communication challenges.
Facial expressions	Lip Corner Tightener (AU14)	5	Facial Action Units (AUs) represent specific facial muscle movements, each corresponding to certain emotions or expressions, such as eyebrow raises, smiles, or frowns. These features measure how frequently and for how long each AU is activated, providing insights into emotional engagement, communication, and non-verbal behavior during interactions. Clinicians can use this information to analyze social responsiveness, emotional regulation, and subtle facial cues.
	Lip Corner Puller (AU12)	5	
	Cheek Raiser (AU6)	5	
	Inner Brow Raiser (AU1)	5	
	Upper Lip Raiser (AU10)	5	
	Smile (AU6+AU12)	3	
Gaze	Eye Contact	11	Frequency and duration of eye contact, key indicators of social engagement in ASD.
	Directed gaze – initiation	5	Measures how often and for how long the individual looks at the wearable camera, which could indicate engagement with the clinician.

This table shows a representative subset of audio-visual features extracted from the audio- and video data.

To account for varying session durations, normalization procedures converted all temporal metrics to event frequency per minute, mean event duration, and percentage of session time. For time-varying features, the pipeline generated session-level metrics through the computation of statistical descriptors (mean, standard deviation, skewness). These normalized metrics comprised the final feature set for classification analysis (**Supplementary Table 3**).

### 2.6 Audio pipeline

Below are the detailed steps of the audio analysis in the Neurora pipeline (**Supplementary Figure 1**). After audio extraction, we employed a noise-filtering model to enhance clarity and address environmental interference, such as echo or background noise (28). This model effectively filters out most of the unwanted noise while preserving the integrity of the speech signal. After noise filtering, we isolated speech segments from non-speech portions, a process known as voice activity detection (29).

To differentiate between speakers in the audio, speech segments transformed into high-dimensional embeddings were clustered based on their similarity (30). Specifically, spectral clustering was used to group these embeddings, with each cluster corresponding to a distinct speaker (30, 31).

The system extracted prosodic features at the utterance level, including temporal modulation spectrum, pitch contours, and speech rhythm patterns (32).

### 2.7 Video pipeline

Below are the details of the video analysis in the Neurora pipeline (**Supplementary Figure 2**). Our 2D pose estimation model is based on a convolutional neural network-based model architecture, which uses nonparametric representation to differentiate body parts of individuals (33).

After the previous steps, head bounding boxes were tracked for each individual. As a next step, faces were detected, and frontal faces were selected. The shape of the face was predicted using the FAN3D model (34). Gaze was estimated using the L2CS-Net model (35). All estimated and tracked individuals were linked together, to have segments of individuals.

For emotion-based metrics, the pipeline also predicts action units, which are used to describe facial muscle movement (36). The pipeline estimates action units (AUs) 1, 6, 10, 12 and 14, respectively. For each action unit a separate model was trained using one or more of the following databases BP4D-SFE (37, 38), DISFA (39), DDCF (40) and CHILDEFES (41). Our AU detection system is based on the emotion classification framework proposed by (42).

### 2.8 Evaluation of validity

Quality control procedures validated all extracted biometric features against manual annotations performed by trained annotators following predefined annotation protocols. The analysis pipeline was validated using manually annotated audio and video segments from a diverse sample of 23 audio and video recordings, which included speech activity, speaker identification, gaze annotations and Action Unit (AU) annotations. The demographic distribution of samples was as follows: 12 males and 11 females, with 15 individuals diagnosed with ASD and 8 identified as NT.

One aspect of the audio analysis quality assessment involved measuring the system’s ability to separate the participant’s voice from other speakers and voice sources. The system automatically separates speakers we call candidates, and manual annotators have confirmed which candidate the participant was before proceeding to the next step in data analysis. Therefore, quantifying quality was reduced to measuring whether candidate speakers contained voice segments from a single speaker, assessed using the purity metric (43) (**Equation 1**).

(1) $$Purity=\frac{\underset{cluster}{\Sigma }\max|cluster\cap spea\ker|}{\underset{cluster}{\Sigma }|cluster|}$$

For the video pipeline, balanced accuracy was calculated to assess the algorithmic quality of each event detector within the pipeline, such as eye contact or AU12 activation. Balanced accuracy (**Equations 2**–**4**) is particularly useful in scenarios with highly imbalanced class distributions, such as in the case of the video pipeline features, where prevalence varies significantly across classes.

(2) $$Balanced\;Accuracy\text{ = }\frac{Sensitivity+Specificity}{2}$$

(3) $$Sensitivity=\frac{True\;Positives}{True\;Positives+False\;Negatives}$$

(4) $$Specificity=\frac{True\;Negatives}{True\;Negatives+False\;Positives}$$

In certain cases, a sample may contain no positive instances, resulting in zero prevalence. Under these conditions, sensitivity becomes undefined, and multiple strategies exist for handling such scenarios. To ensure clarity, we report these samples separately and use specificity as the performance metric in place of balanced accuracy.

To establish ground truth annotations, trained annotators followed a standardized protocol and manually identified vocal activity behaviors using a specialized user interface. Gaze annotation ground truth was created using a standardized protocol as well. AU ground truth was created by annotators with Facial Action Unit Coding (FACS) certification. These annotations served as the reference for evaluating the outputs of the analysis pipeline. Multiple annotators took part in the process and a subset of the samples have annotations from more than one annotator, so we could establish inter-rater reliability using Cohen’s Kappa (**Supplementary Table 5**) (44, 45). The results of the validity evaluation are presented in the online **Supplementary Material** (eResults).

### 2.9 Machine learning diagnosis classification

To account for differences in verbal ability across ADOS-2 modules, the participants were divided into three groups: few-to-no-words (Module T and 1), phrase speech (Module 2), and fluent speech (Module 3). This approach ensured that diagnostic features were learned and evaluated within comparable developmental contexts (46, 47).

Random Forest classifiers were developed to distinguish ASD from non-ASD (clinical and NT) participants based on reference standard diagnoses, using 97 biometric features extracted from the ADOS-2 multimodal recordings through the analysis pipeline, alongside participant chronological age and sex as input variables. Random Forest was selected due to its effectiveness with high feature-to-sample ratios and non-normal feature distributions (48). The derived features showed high cross-correlation, which does not influence the overall performance of Random Forests, but limits the interpretability of feature importance (49), a calculated trade-off of this method. To achieve a comprehensive evaluation of the classification method given the sample sizes, the dataset was split into an inner set and hold-out test set, and quality metrics of the results were acquired for both sets. Hold-out participant selection was stratified to maintain balance across diagnostic classifications and cohorts.

The classification pipeline (see **Supplementary Figure 4**) was evaluated using leave-one-out cross-validation (LOO-CV) on the inner set, training N models on N-1 samples each, and testing each model on a corresponding single held-out sample (50). To address class imbalance, Balanced Bagging was applied (51), selecting 10 random balanced subsets of the data for training separate submodels for each LOO-CV fold. The number of decision trees was optimized via grid search; a nested 5-fold cross-validation where the competing models were scored based on their accuracies. Each Random Forest produced probability estimates as classification outcomes, which were aggregated across submodels of the Balanced Bagging classifier. The decision threshold was tuned to account for a minimal difference between sensitivity and specificity using the prediction outcomes for each sample in the entire inner set, irrespective of the language/developmental level (**Supplementary Figure 5**). Performance evaluation encompassed sensitivity, specificity, accuracy, balanced accuracy, positive predictive value and negative predictive value. Confidence intervals for each of these metrics were estimated using 1000 randomly resampled prediction sets. To assess whether the models produced statistically distinct probability distributions between diagnostic groups, Mann-Whitney-Wilcoxon tests were performed comparing the probability outputs for ASD versus non-ASD participants within each group. All prediction outputs are concatenated into a unified list of predictions before calculating the above listed metrics, aggregated across language and developmental level groups.

To provide an additional assessment of generalizability, a final model was fitted to the whole inner set using the above pipeline, with the decision threshold calculated from the cross-validation results. Its performance was evaluated on the hold-out test set.

## 3 Results

Of the 546 participants enrolled across sites, 458 (83.6%) met quality indicators for both video and audio recordings and comprised the primary analysis set. The mean chronological age of the participants was 79.04 ± 40.97 months, with n=166 (36.2%) female and n=292 (63.8%) male participants (**Table 1**, **Supplementary Table 4**). Statistical relevance of the results was further confirmed, to explore distributional differences in the model confidence, predicted probability estimates were grouped by ground-truth class (ASD vs. Non-ASD), and a Mann-Whitney-Wilcoxon rank sum test was conducted (52), to assess whether the distributions differ significantly at the given sample sizes (**Supplementary Table 10**). Feature importance analysis indicated that the predictive signal was distributed across multiple behavioral features, with all individual feature importance values remaining below 1%, suggesting no single dominant predictor. To assess whether stratifying models by developmental levels improves classification performance, we compared models on the pooled populations with those trained separately for each subgroup (**Supplementary Table 14**). The joint model showed reduced discriminative ability as it failed to adequately distinguish between younger non-ASD from older ASD subjects.

### 3.1 Performance on the inner- and hold out sets

**Figure 3:**
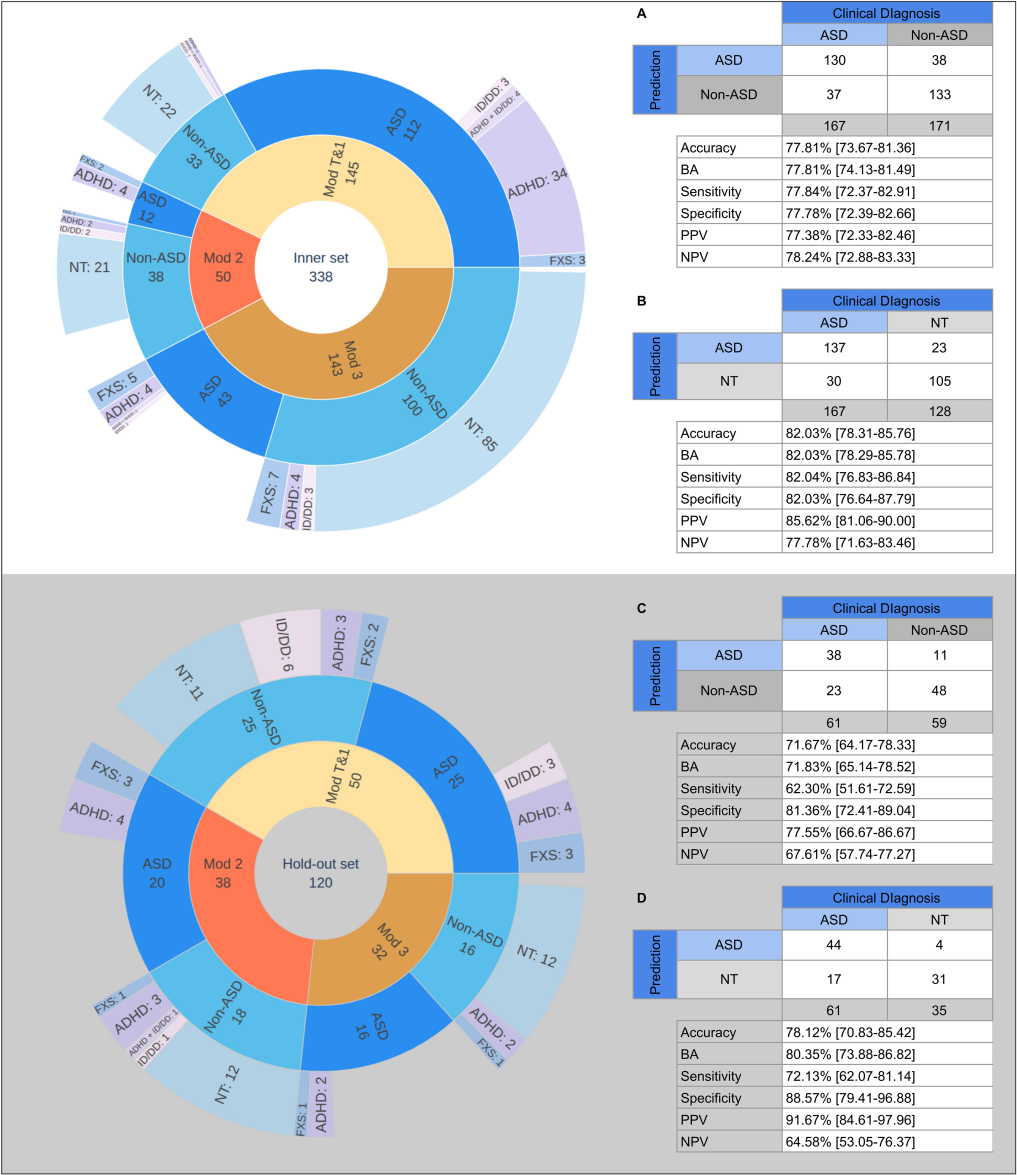
Sunburst charts display the hierarchical distribution of participants by ASD diagnosis (inner ring), ADOS-2 module (middle ring) and further diagnosis (outer ring, NT=neurotypical, FXS=fragile X syndrome, ID/DD=intellectual disability or developmental delay, ADHD=attention-deficit/hyperactivity disorder). ADOS-2 modules denote the different language and developmental level groups (few-to-no words group - Mod T/1, phrase speech group - Mod 2, fluent speech group - Mod 3). Confusion matrices and performance metrics are displayed on the right: **(A)** Trained and evaluated using leave-one-out cross-validation (LOO-CV) on the full inner set cohort (n=338); **(B)** LOO-CV-trained on the full inner cohort, evaluated with the exclusion of the participants with non-ASD clinical conditions (n=43); **(C)** trained on the full inner cohort, tested on the hold-out set (n=120); **(D)** trained on the full inner cohort, tested on the hold-out set with the exclusion of participants with non-ASD clinical conditions (n=24) Performance metrics include overall accuracy, balanced accuracy (BA), sensitivity, specificity, positive predictive value (PPV), and negative predictive value (NPV). Values in parentheses indicate 95% confidence intervals. The experimental setup is shown in **Supplementary Figure 4** of the **Supplementary Material**.

**Figure 3** presents the aggregated results for the main analysis of ASD (idiopathic and syndromic) and non-ASD (clinical and NT). On the inner set and across all groups (n=338), 77.8% sensitivity and specificity were achieved for distinguishing ASD from non-ASD participants using biometric data (**Figure 3A**). When the model was evaluated for differentiating between ASD and NT participants only, sensitivity and specificity increased to 82.0% (**Figure 3B**). Based on the results of the inner set (**Figure 3A**; **Supplementary Tables 7**, **8a, b**, **10**, **12**), the model achieved a 53% classification accuracy for the non-ASD clinical subgroup (n=43, 23 classified correctly, **Supplementary Table 10**) and 85.9% accuracy for the non-ASD NT subgroup (n = 128, 110 classified correctly, **Supplementary Table 10**).

The hold-out test set (n=120) demonstrated wider confidence interval ranges than the inner set due to its smaller sample size. Sensitivity decreased moderately compared to the inner set for ASD/non-ASD differentiation, while specificity remained mostly unchanged. Results for the hold-out test set showed 62.3% sensitivity and 81.4% specificity for ASD versus non-ASD participants (**Figure 3C**) with 72.1% sensitivity and 88.6% specificity when differentiating between the ASD and NT subgroups (**Figure 3D**). The model achieved a 62.5% classification accuracy for the non-ASD clinical subgroup (n=24, 15 classified correctly, **Supplementary Table 10**) and 94.3% accuracy for the NT subgroup (n = 35, 33 classified correctly, **Supplementary Table 10**).

### 3.2 Performance by developmental level

In the case of the inner set, results of the models for distinguishing between ASD and non-ASD by ADOS-2 module levels are as follows: few-to-no words (87% sensitivity, 58% specificity), phrase speech (67% sensitivity, 89% specificity) and fluent speech (58% sensitivity, 80% specificity). The few-to-no-words model showed a higher sensitivity for the more populated Module 1 (88%), while specificity is much higher for the toddlers (81%, **Supplementary Tables 8a, b**). If the model had to distinguish between ASD and NT by the developmental levels, the results were as follows: few-to-no words (89% sensitivity, 73% specificity), phrase speech (75% sensitivity, 90% specificity) and fluent speech (65% sensitivity, 82% specificity)(**Supplementary Tables 8a, b**).

### 3.3 Performance by sex

Performance in the inner set varied by demographics, with females showing 75% sensitivity and 84% specificity, while males showed 79% sensitivity and 70.0% specificity. Performance on the hold-out set presented a similar pattern as females showed 60% sensitivity and 83% specificity, while males showed 63% sensitivity and 80% specificity in the case of the classification between the ASD and non-ASD groups. The subgroup based analysis is limited by the sample size; these results are presented in **Supplementary Tables 8a, b**, **9a, b** of the **Supplementary Material** for reference.

### 3.4 Performance by co-occurring conditions

Participants diagnosed with co-occurring conditions such as FXS, ADHD, and ID/DD were distributed across ASD and non-ASD groups (**Figure 3**). Model performance for co-occurring condition across all available participants (aggregated results from the inner set and hold out set, **Supplementary Table 12**) showed: ADHD (ASD = 57, non-ASD=17) achieved 84% sensitivity and 59% specificity; ID/DD (ASD = 12, non-ASD=15) showed 67% sensitivity and 47% specificity; FXS (ASD = 17, non-ASD=12) showed 94% sensitivity and 50% specificity.

### 3.5 Performance by site

In the case of the inner set, Qatar participants achieved 86% sensitivity and 77% specificity. This is compared to 62% and 78% for the US participants for the ASD/non-ASD classification, noting the higher and exclusive prevalence of FXS participants in the US collection (**Supplementary Table 13**). The Qatari cohort (n=187) demonstrated 10% higher accuracy and 11% higher balanced accuracy than the US cohort (n=151) (**Supplementary Table 7**). In the case of the ASD/NT classification, sensitivity was higher in both sites (from 62% to 69% in the US and from 86% to 89% in Qatar), while specificity increased in the case of Qatar (from 77% to 92%) and slightly decreased in the case of US data (78% to 76%) (**Supplementary Tables 8a, b**, **13**). In the case of the hold out set these trends were mostly the same, but the classification of the US data provided better sensitivity (68%) compared to the Qatar cohort (57%) (**Supplementary Table 13**).

## 4 Discussion

In this multicenter, multinational study, we demonstrated that automated behavioral analysis during standardized clinical assessments can provide objective diagnostic support while maintaining naturalistic testing conditions. The proposed methodology analyzed audio-visual biometric data of social-communication behaviors during ADOS-2 administrations and supported autism diagnosis in a phenotypically and ethnically diverse sample of children aged 2–12 years. Training the models within the semi-structured observational contexts of ADOS-2 Modules T, 1, 2, and 3 supported developing a tool with capacity to support participants with differing language and developmental profiles, providing utility across a broad range of ASD and within a common clinical framework (6). The system achieved 77.8% sensitivity and 77.8% specificity on the inner set using LOO-CV-evaluation, and 62.3% sensitivity and 81.4% specificity on the hold-out test set comparing autism (idiopathic and syndromic) to non-ASD (clinical and NT) populations.

The output of the pipeline includes the extracted features including paralinguistic metrics, vocalizations, facial expressions, and gaze that underlie the predictive model (**Table 2**). By quantifying these features, the system provides clinicians with objective behavioral metrics of subtle social communication behaviors with potential to complement clinical judgement. Some features (**Table 2**; **Supplementary Table 3**) would be difficult for all but the most highly trained clinicians to detect and impossible to quantify independently. Subtle features such as spectral features for vocalization (32) and interactive gaze elements have potential to provide nuanced clinical and phenotypic data informing research and practice. Importantly, the proposed system has yet to capture several key behavioral features in autism, namely gestures and restricted and repetitive behaviors. Thus, applications of the tool (like many clinical tools) are inherently geared to inform, quantify, and support clinical workflows seeking task-sharing and deliver nuanced social communication data profiles.

Detection rates from this tool are comparable to rates reported in recent meta-analyses for single-modality, computational autism diagnostic tools such as eye-tracking and neuroimaging, though without the requirements for fixed position, experimental tasks (17, 18, 53, 54). For a detailed comparison see **Table 3**. Additionally, data were collected during standardized clinical evaluations without hardware or restrictions on participants, allowing clinicians to utilize the technology within routine clinical workflows. To our knowledge this study represents the most diverse, representative sample in autism digital health diagnostic research with respect to age, developmental levels, verbal abilities, clinical profiles, language and cultural backgrounds (e.g., US and Qatar) (**Table 1**; **Supplementary Table 5**).

**Table 3:** Overview of reported performance metrics from selected computational approaches for autism classification across different datasets and methodologies.

Study	Dataset	Sensitivity	Specificity	Features	n	Age range
Current study	Inner set ASD–non-ASD results	77.84% (95% CI: 72.37–82.91%)	77.78% (95% CI: 72.39-82.66%)	social communication - paralinguistic metrics, social communication – vocalization, facial expressions, gaze	338	12–155 months old
Current study	Inner set ASD–NT results	82.04% (95% CI: 76.83–86.84%)	82.03% (95% CI: 76.64–87.79%)			
Current study	Hold-out test set ASD–non-ASD results	62.30% (95% CI: 51.61–75.59%)	81.36% (95% CI: 72.41–89.04%)		120	
Current study	Hold-out test set ASD–NT results	72.13% (95% CI: 62.07–81.14%)	88.57% (95% CI: 79.41–96.88%)			
Megerian et al., 2022 (17)	Test Overall	51.64% (95% CI: 42.4%–60.8%)	18.48% (95% CI: 14.3%–23.3%)	caregiver questioner, manual home video analysis, healthcare provider questioner	425	18–72 months old
Megerian et al., 2022 (17)	Determinate category analysis	98.44% (95% CI: 91.6%–99.96%)	78.87% (95% CI: 67.56%–87.67%)		145	
Jones et al., 2023 (18)	Test Overall	71% (95% CI: 64.6%–76.9%)	80.7% (95% CI: 75.3%–85.4%)	social visual engagement via eye-tracking	475	16–30 months
Jones et al., 2023 (18)	Test certain diagnosis only	78.0% (95% CI: 70.5%–84.3%)	85.4% (95% CI: 79.5%–90.2%)		335	

For reference, data from Megerian et al., 2022 (17) and Jones et al., 2023 (18) was added. The table summarizes reported sensitivity and specificity values from the respective studies on their own evaluation datasets. Megerian et al., 2022 (17) - Determinate category analysis included participants where the device rendered a determinate output. Jones et al., 2023 (18) – Test certain diagnosis only included participants with certain clinical diagnosis. ASD, Participant with ASD; non-ASD, Neurotypical and non-ASD clinical participants; NT, neurotypical.

When distinguishing ASD from NT participants, the performance gap between inner and hold-out test set narrows (regarding the classification sensitivity, there is 15.5% difference between ASD and non-ASD in the case of the inner set and 9.91% difference between ASD and NT in the case of the hold out set). This study also sought to apply computational models to symptom profiles in common co-occurring conditions such as those represented in the non-ASD clinical group (e.g., genetic syndromes, ADHD). Challenges with differential diagnosis between the ASD and non-ASD groups are reflected in the decreased sensitivity from the inner set (77.8%) to the hold-out test set (62.3%), although specificity was maintained (77.8% to 81.4%). In the hold-out set, lower classification efficiency for comorbidities has a greater effect on aggregated metrics due to the smaller total number of participants. These data are consistent with data from application of standard diagnostic tools to psychiatrically and medically complex populations (55, 56). For example, the ADOS-2 achieves 58.3% sensitivity and 56.5% specificity (57) in children with autism and comorbid psychiatric disorders. Similar studies underscore the need for careful consideration when utilizing existing cutoff scores and guidelines (58), and highlight the value of extended evaluations and additional measures (59). In our models, ADHD cases (n=69) showed 84% sensitivity but only 59% specificity, while fragile X syndrome demonstrated 94% sensitivity with 50% specificity.

In exploratory analyses of the system across developmental levels, data revealed important considerations of language and developmental levels. The models maintained moderate ability to discriminate between diagnostic groups despite the low sample numbers (Mann-Whitney-Wilcoxon p<0.001 for all examined language and developmental level groups). The few-to-no words model achieved 87% sensitivity but only 58% specificity, while the phrase speech and fluent speech groups showed opposite patterns (75% and 64% sensitivity with 89% and 83% specificity). These results likely reflect the higher proportion of autism cases in the Module 1 cohort of the few-to-no-words group and the relatively easier task of autism diagnoses in younger children (60). To further verify the benefit of stratified modeling by developmental level, we compared pooled and stratified models (**Supplementary Table 14**), which showed that the joint model struggled to distinguish younger non-ASD from older ASD participants, validating our assumption that diagnostic features should be learned and evaluated within comparable developmental contexts.

Differences between the international cohorts reported in this study appear to be the result of differences in clinical recruitment strategies. That is, the performance difference between Qatar (86% sensitivity) and US sites (62% sensitivity) may be explained by higher autism prevalence in the Qatar cohort (56.3% vs 42.2%) and the targeted recruitment of fragile X syndrome in the US cohort (n=29), which showed notably low specificity (50%). In the case of both cohorts, accuracy and balanced accuracy was higher in the case of ASD-NT classification. The improvement was primarily driven by the increased specificity in the case of the data from Qatar (77% to 92%) and by the increased sensitivity in the case of the data from the USA (62% to 69%) compared to the ASD-non-ASD classification (**Supplementary Table 13**). While multi-national digital health analytic approaches are needed, further efforts are required to understand potential cultural variables influencing diagnosis and monitoring.

Overall, this study demonstrates the feasibility of a semi-automated multimodal computational analysis for supporting autism assessment within naturalistic clinical settings, including pediatric evaluations characterized by complex and dynamic interaction patterns. While not intended to replace clinical judgment, the proposed system provides objective quantification of social-communication behaviors that may complement existing diagnostic practices, with the potential to improve consistency and reduce assessment burden. This work establishes initial evidence for clinical relevance and feasibility of the proposed pipeline as a task-sharing tool, creating a basis for future investigations across a broader range of clinical applications. In the context of ADOS-2 and other specialized observational assessments, the computational approach has potential to distribute and expedite standard workflows by separating administration (e.g., by technicians, therapists) and coding/scoring/clinical decision making (e.g., diagnosticians). The standardized representation of behavior through multiple quantified features, also provides more consistent interpretation of the observations during the diagnostic process, reducing the variability and supporting objective multimodal measurements with potential to supplement longitudinal follow-up.

While this study highlights detection capacities of the model, the emphasis on multi-modal social communication metrics enhances future potential to utilize the tool to support both diagnosis and monitoring of symptom trajectories over time. To date, few structured observational tools (clinical or digital) - provide support for treatment tracking. One such specialized observational tool, the Brief Observation of Social Communication Change (BOSCC) (45) aims to distill key autism social-communication behaviors into structured, reviewable output from brief parent-child interactions. It is typically scored from video by trained, independent raters distinct from the administering clinician, providing blinded, objective and scalable behavioral assessment for monitoring in clinical and research settings (61). Models outlined in this study provide similar, potentially complementary digital approaches with capacity for rapid capture of nuanced social communication behaviors such as initiation and frequency of gaze, facial expressions and spectral features of vocalizations.

## 5 Limitations and future work

Several limitations of this study should be acknowledged. Even though the ADOS-2 provides a well-established and standardized context for eliciting clinically relevant social-communication behaviors, reliance on a single methodology constrains the generalizability of findings. The study also lacks IQ data from Qatar, however the system responded well across developmental domains regardless. Consequently, the performance and applicability of the proposed approach must be evaluated on data collected under less standardized conditions in future work. Since only participants who met the quality threshold for both audio and video recording were included, exclusions due to poor video or audio quality highlight ongoing technical challenges in capturing naturalistic data within real-world clinical environments. Additionally, the Tobii glasses capture AUs only when the clinician is facing the child. Future work will aim to extend the included domains (e.g. repetitive behaviors). Although the study leveraged a diverse, multi-site dataset, sample sizes remain a limitation, particularly within demographic and diagnostic subgroups. While the proposed approach demonstrates promise for augmenting clinical diagnosis, differential diagnosis remains challenging, in part because the number of participants with co-occurring clinical conditions was low. Future work will focus on expanding the dataset with such cases to enhance the differentiating capacity, as well as extending the range of captured behavioral domains, such as repetitive behaviors. It is also important to note that short-timescale co-occurrence between behavioral domains are important. Long-term temporal structure also contains valuable information, however, it is methodologically more challenging in the ADOS-2 context, as task order and interaction flow can vary across administrations. Future work will focus on the integration of temporal and sequential features as an important next step.

## 6 Conclusion

In summary, the classification performance observed in this study supports the feasibility of multimodal social-communication behavioral feature extraction designed to operate within naturalistic clinical environments. Future work will aim to extend validation to additional diagnostic frameworks, diverse data sources, and non-specialist clinical settings, with the goal of improving consistency, sensitivity and characterization in complex populations, and generalizability across settings (e.g., culture, clinics).

## Data Availability

The raw data supporting the conclusions of this article will be made available by the authors, without undue reservation.
