# Supplementary material for: A naturalistic, non-invasive method for capturing biometric data during autism evaluations
Source: Front Psychiatry. 2026 Jun 11;17:1819384. doi: 10.3389/fpsyt.2026.1819384 (PMC13294302; doi:10.3389/fpsyt.2026.1819384)
Supplement: Supplementary file 1 [file DataSheet1.pdf]

Supplementary Material

1. Supplementary Data

1.1. Evaluation of Validity

The reliability of manual annotation using Cohen's Kappa shows "Almost perfect agreement" for three of the analyzed event types and "Substantial agreement" for the AUs, which shows the difficulty of AU annotation even for trained humans. We have measured the quality of speaker diarization in the audio pipeline with the purity metric, because in our pipeline annotators select the Patient from speaker candidates and validity depends on these candidate speakers containing vocalization segments truly coming from a single speaker. We have used 0.25 second collar similarly to others in the literature (1).

An average of 0.87 purity results show that the candidate speakers identified in our system contain segments mostly associated with a single speaker, allowing the annotators to correctly identify the Patient speaker from them.

For the patient looking into the eye region of the clinician prediction, the video pipeline could achieve an average balanced accuracy of 0.73. These results are on par with recent results in a similar environment (2). For the clinician looking into the eye region of patient prediction, we could achieve average balanced accuracy of 0.92 and eye contact, which is an intersection of the "Looking into camera" and "Looking at patient" events, had an average balanced accuracy of 0.8. For the three cases, denoted in the table, where prevalence was 0 our model performed with a specificity of 1 (no false positives predicted).

The overall performance of the AU detection model on this small evaluation set is limited, with balanced accuracy values averaging around 0.6 aggregating the different AUs, with AU6 emerging as the best performing one. Several factors likely contribute to this outcome. First, currently periods of speech are not accounted for in the model, while they have an effect on AU manual annotation in line with the FACS standards. Additionally, although not yet systematically analyzed, it is plausible that the system confuses similar AUs. Such misclassifications are currently treated as errors in the evaluation metrics, rather than being recognized as confusion between related units. We have filtered 10 samples with prevalence=0, the mean specificity for them is 0.83, showing a limited number of false positives in these cases. Nonetheless, despite these limitations, the metrics derived from this AU event detector have played a significant role in diagnosis classification, as evidenced by the prominence of AU6, AU12, and happiness in the feature importance reports.

2. Supplementary Figures and Tables

2.1. Supplementary Figures

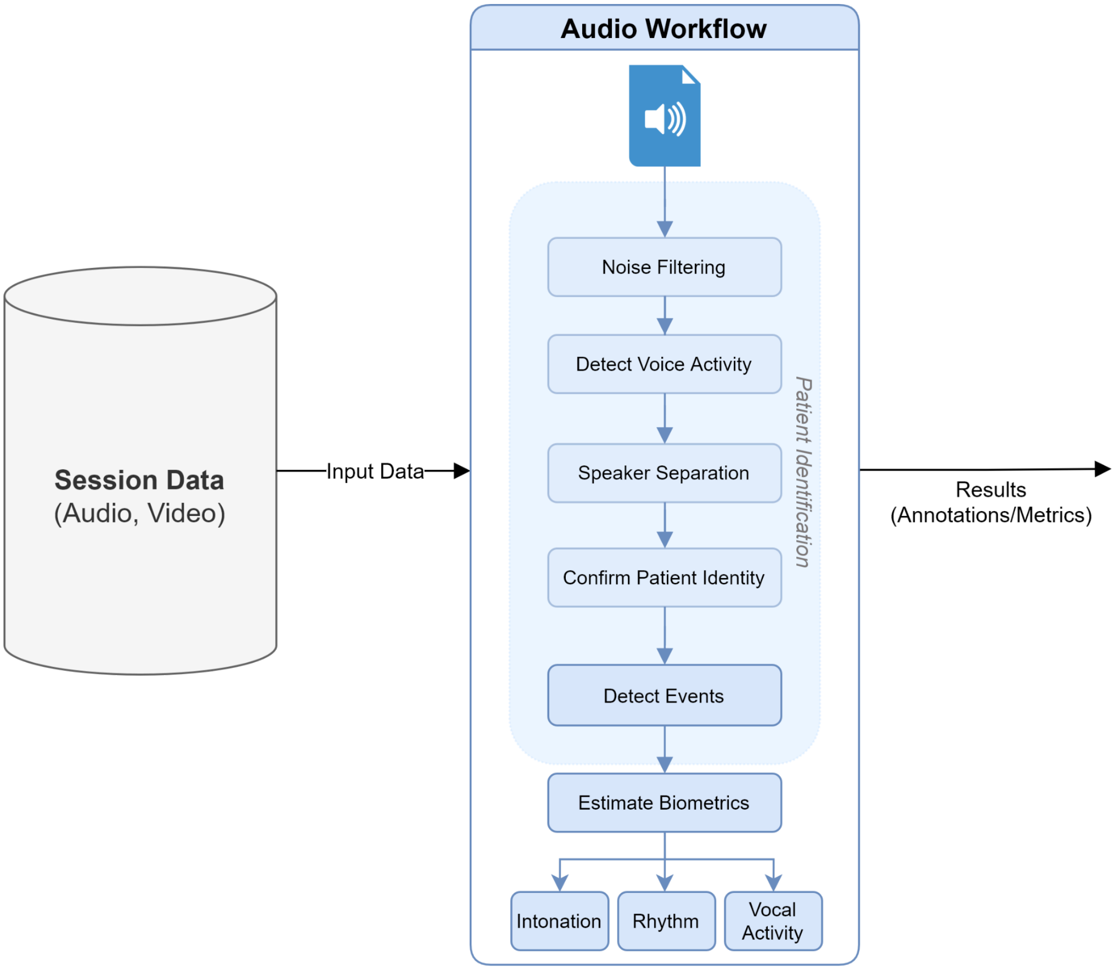

Supplementary Figure 1: Audio data processing pipeline. Flow of the datastream in the analysis pipeline.

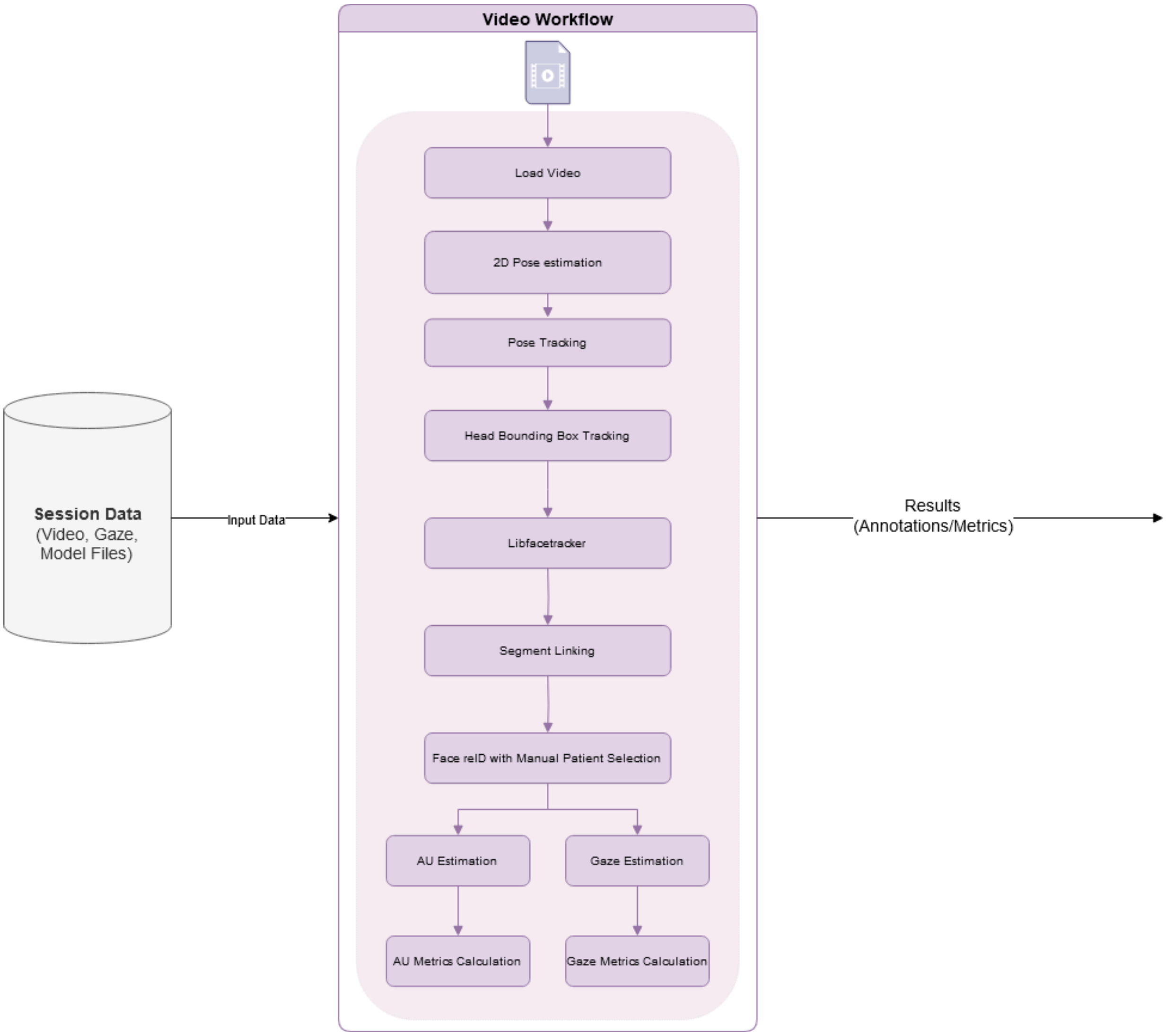

Supplementary Figure 2. The video data processing pipeline. The datastream in the analysis pipeline from loading the input video to providing the biometrics.

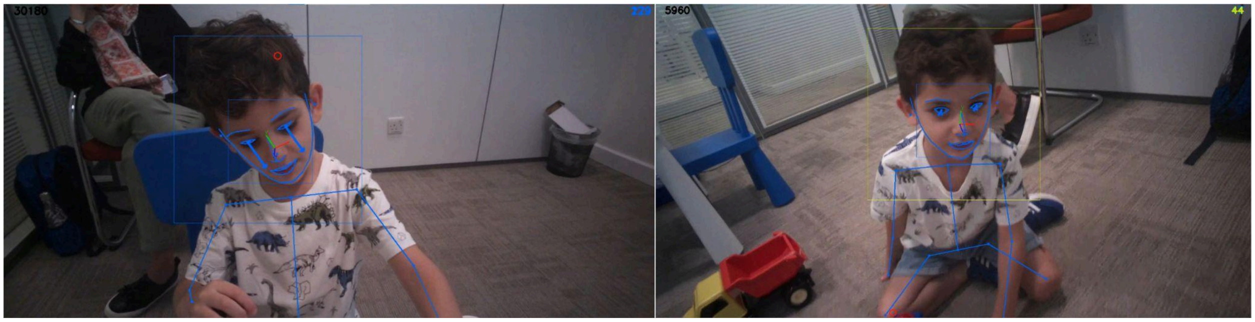

Supplementary Figure 3. Visualization of the features detected on the video.

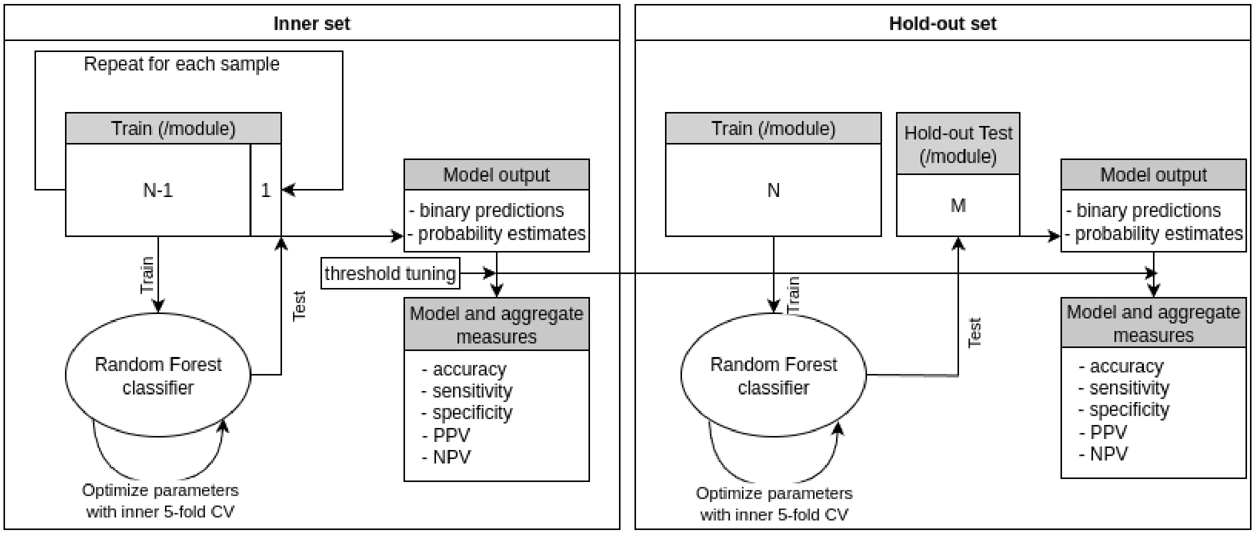

Supplementary Figure 4. Experimental setup for the model training and testing.

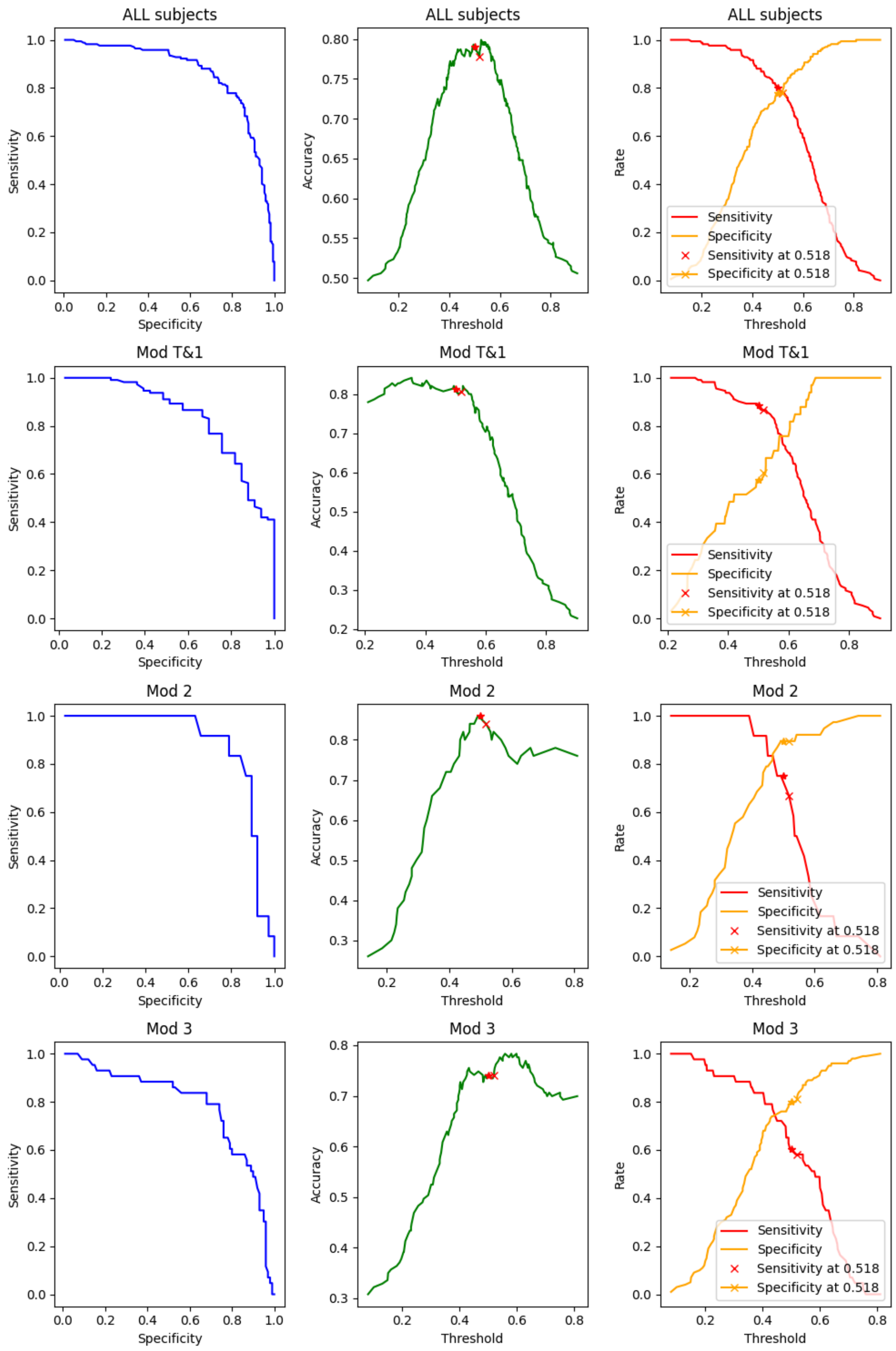

Supplementary Figure 5. Calibration plots for the decision threshold for tuned classification in the case of the ASD-non-ASD classification in the case of the inner set. The threshold was calculated for the aggregated set, its module-wise influence is shown for reference. On the

middle graphs, X denotes the tuned decision threshold, while * denotes the 0.5 decision threshold.

2.2. Supplementary Tables

Overall	Qatar N=300 (54.6%)	US N=248 (45.3%)
**Female**	N=95 (17.34%)	N=108 (19.71%)
**Male**	N=205 (37.41%)	N=140 (25.55%)
**Autism**	N=164 (29.93%)	N=101 (18.43%)
**Non-autism Clinical**	N=53 (9.67%)	N=26 (4.74%)
**Neurotypical**	N=83 (15.15%)	N=121 (22.08%)
**Fragile X Syndrome**	N=0 (0%)	N=38 (6.93%)
**ADHD**	N=83 (15.15%)	N=0 (0%)
**Intellectual Disability/ Developmental Delay**	N=26 (4.74%)	N=9 (1.64%)

Supplementary Table 1. Sex and diagnostic distribution of the enrolled subjects based on country of recruitment.

DIAGNOSTIC GROUPS	CLINICAL DIAGNOSIS	NUMBER OF SUBJECTS
ADHD	ADHD	89
Endocrine Disease	DIABETES	1
Epilepsy and (other) Seizure Disorder	EPILEPSY	13
	SEIZURES DISORDER	1
Genetic Disorder	FXS	38
	CAMK2A GENE MUTATION	1
	DOWN SYNDROME	3
	DRAVET SYNDROME	1
	GENE 13 DUPLICATION	1
	GENETIC ABNORMALITY (WASF1 GENE)	1
	HEXOGENOME STUDY: RARE MUTATION CAUSING HYPOXIC LESION	1
	KABUKI SYNDROME	1
	MICROCEPHALY	1
	NF1 GENE MUTATION	2
	PMS	13
	SOTOS SYNDROME	1
Intellectual Disability/ Developmental Delay	CHILD DEVELOPMENT DISORDER	1
	DELAYED COGNITIVE ABILITY	1
	DEVELOPMENTAL DELAY	2
	GDD	2
	ID	19
	LANGUAGE DELAY	1
	MILD RETARDATION	1
	SPEECH DELAY	19
	WEAK COMMUNICATION SKILLS	1
(Other) Medical Comorbidities	PHYSICAL DISABILITY	1
	SENSORY PROCESSING DISORDER	2
	ANXIETY DISORDER	6
	LEARNING DISORDER	4
	SOCIAL SKILL DELAYS	1
	TICKS	1

Supplementary Table 2. The distribution of non-autism conditions of the enrolled subjects during the study. One subject might have multiple comorbidities simultaneously

Feature Category	Specific Features	Purpose	Statistical Descriptors	References
Voice Features	Pitch (F0), Envelope Spectrum, Intrinsic Mode Functions, Temporal Modulation Spectrum	Capture vocal patterns and acoustic characteristics, providing insights into speech rhythm and pitch variation.	Mean, SNT Dev, Skewness, Kurtosis, Min/Max, etc.	Lau et al. (3)
Gaze features	Looking into Camera<br>Looking at Person<br>Eye contact	Analyze the gaze of the patient and the eye contact between the patient and clinician	Count, Duration, Rate, Mean duration	Stuart et al. (4)
Action Units	AU1<br>AU6<br>AU10<br>AU12<br>AU14	Analyze facial action units, which encodes facial muscle movement.	Count, Duration, Rate, Mean duration	Begeer et al. (5), Trevisan et al. (6)

Supplementary Table 3: Extracted features of the audio- and video pipeline with the purpose of the feature, the statistical descriptors and the references.

All participants (N = 458)	(US = 211, Qatar = 247)	Autism (N=228)				Non-Autism (N=230)			
		Module T	Module 1	Module 2	Module 3	Module T	Module 1	Module 2	Module 3
**Count**		14	123	32	59	31	27	56	116
**Age**	Mean (SD)	23 (3.23)	71.56 (37.07)	96.13 (38.05)	122.71 (26.72)	21.77 (5)	57.3 (32.34)	55.38 (22.57)	95.53 (28.55)
	Median	23.0	57.0	96.5	119.0	22.0	43.0	51.0	94.5
**Sex, No. (%)**	Female	2 (14.29)	26 (21.14)	6 (18.75)	13 (22.03)	14 (45.16)	10 (37.04)	25 (44.64)	70 (60.34)
	Male	12 (85.71)	97 (78.86)	26 (81.25)	46 (77.97)	17 (54.84)	17 (62.96)	31 (55.36)	46 (39.66)
**Race, No. (%) ***	American Indian or Alaska Native	0 (0)	2 (1.63)	0 (0)	0 (0)	0 (0)	0 (0)	0 (0)	0 (0)
	Asian	0 (0)	1 (0.81)	1 (3.13)	1 (1.69)	0 (0)	0 (0)	0 (0)	0 (0)
	Black or African-American	1 (7.14)	4 (3.25)	4 (12.5)	4 (6.78)	5 (16.13)	1 (3.7)	2 (3.57)	14 (12.07)
	White	3 (21.43)	8 (6.5)	7 (21.88)	9 (15.25)	15 (48.39)	1 (3.7)	11 (19.64)	47 (40.52)
	More than one race	0 (0)	1 (0.81)	0 (0)	3 (5.08)	0 (0)	0 (0)	1 (1.79)	3 (2.59)
	Unknown or not reported	10 (71.43)	107 (86.99)	20 (62.5)	42 (71.19)	11 (35.48)	25 (92.59)	42 (75)	52 (44.83)
**Ethnicity, No. (%) ****	Hispanic or Latino	2 (14.29)	7 (5.69)	5 (15.63)	5 (8.47)	7 (22.58)	0 (0)	3 (5.36)	15 (12.93)
	Not Hispanic or Latino	3 (21.43)	9 (7.32)	9 (28.13)	11 (18.64)	13 (41.94)	2 (7.41)	11 (19.64)	47 (40.52)
	Unknown or not reported	9 (64.29)	107 (86.99)	18 (56.25)	43 (72.88)	11 (35.48)	25 (92.59)	42 (75)	54 (46.55)
**ADOS-2 Social Affect Score**	Mean (SD)	15.86 (4.16)	14.46 (3.99)	10.5 (4.3)	9.03 (3.85)	4.58 (3.96)	5.11 (4.6)	2 (2.41)	2.07 (2.44)
	Median	17.0	16.0	10.5	8.0	4.0	4.0	1.0	1.0
**ADOS-2 RRB Score**	Mean (SD)	3.93 (1.91)	4.16 (2.1)	3.22 (2.23)	2.85 (1.96)	0.87 (1.26)	1.15 (1.27)	0.68 (1)	0.63 (1)
	Median	4.0	4.0	3.0	3.0	0.0	1.0	0.0	0.0
**ADOS-2 Total Score**	Mean (SD)	19.86 (5.64)	18.62 (4.81)	13.72 (5.72)	11.88 (4.8)	5.45 (4.73)	6.26 (4.82)	2.68 (3.02)	2.7 (2.88)
	Median	21.5	19.0	12.5	12.0	5.0	1.5	2.0	5.0

**ADOS-2 Comp. Score**	Mean (SD)	-	6.73 (1.65)	6.44 (1.89)	6.8 (2.33)	-	2.37 (1.57)	1.75 (1.35)	1.86 (1.5)
	Median	-	7.0	6.0	7.0	-	2.0	1.0	1.0
**Vineland Adaptive Behavior Scale-3 *****	Adaptive Behavior Composite, mean (SD)	75.5 (7.31)	56.17 (11.43)	67.1 (12.79)	79.66 (20)	101 (14.8)	63.5 (15.93)	88 (22.49)	100.93 (15.93)
**Country of data collection, No.**	US	6	21	14	47	20	9	19	76
	Qatar	8	102	18	11	11	20	36	41

Supplementary Table 4. Participant Characterization and Demographics Note by ADOS-2 Modules. The Autism Diagnostic Observation Schedule, Second Edition (ADOS-2) includes the Social Affect and Restricted and Repetitive Behavior (RRB) domain scores. The Motor domain of the Vineland-3 was administered to participants under 10 years of age. * Race metadata was only available for RUSH samples. ** Ethnicity metadata was only available for RUSH samples. *** RUSH used different versions of Vineland in different phases (version 2 and 3), so the statistics are calculated based on the results of both types of assessment.

Event type	Cohen's Kappa [Mean±SNT, n]
Vocalization	0.885±0.037, n=10
Patient looking into the Clinician's eye region	0.875±0.041, n=4
Clinician looking into the Patient's eye region	0.821±0.076, n=4
Action Units	0.752±0.113, n=10

Supplementary Table 5. Cohen's kappa values to quantify interrater reliability.

Metric	Speaker diarization Purity (collar=0.25 sec)	Looking into camera Mean Balanced Accuracy	Looking at patient Mean Balanced Accuracy	Eye contact Mean Balanced Accuracy	AU1 Mean Balanced Accuracy	AU6 Mean Balanced Accuracy	AU10 Mean Balanced Accuracy	AU12 Mean Balanced Accuracy	AU14 Mean Balanced Accuracy
**All (n=23)**	0.87	0.767	0.92*	0.8**	0.59**	0.70***	0.58***	0.63	0.57**

Supplementary Table 6-a. Analytical validation of the different modules in the analysis pipeline, using the validation dataset (n=23).

Metric	ModT (n=6)	Mod1 (n=6)	Mod2 (n=3)	Mod3 (n=8)
Speaker diarization Purity (collar=0.25 sec)	0.85	0.77	0.89	0.96
Looking into camera Mean Balanced Accuracy	0.767	0.887	0.909	0.864
Looking at patient Mean Balanced Accuracy	0.94*	0.97	0.94	0.89
Eye contact Mean Balanced Accuracy	0.745**	0.735	0.91	0.85
AU1 Mean Balanced Accuracy	0.55*	0.57	0.55	0.62*
AU6 Mean Balanced Accuracy	0.70	0.69*	0.71*	0.70*
AU10 Mean Balanced Accuracy	0.56**	0.62	0.51	0.62*
AU12 Mean Balanced Accuracy	0.65	0.57	0.62	0.67
AU14 Mean Balanced Accuracy	0.62	0.54	0.66*	0.52*

Supplementary Table 6-b. Analytical validation of the different modules in the analysis pipeline, using the validation dataset (n=23). Purity was used for the speaker diarization module. In the case of the other modules, Mean Balanced Accuracy was used for samples with prevalence >0. If prevalence is zero, there is virtually no event present in the two minutes segment. * notation means how many samples from all were not used for the calculation because they had prevalence=0.

Group	N_{ASD}	$N_{non-ASD}$	Accuracy [95% CI]	BA [95% CI]	Sensitivity [95% CI]	Specificity [95% CI]	PPV [95% CI]	NPV [95% CI]
**Aggregated tuned classification CV metrics**								
ASDvsNon-ASD	167	171	0.78 [0.74-0.81]	0.78 [0.74-0.82]	0.78 [0.72-0.83]	0.78 [0.72-0.83]	0.77 [0.72-0.82]	0.78 [0.73-0.83]
ASDvsNT *	167	128	0.82 [0.78-0.86]	0.82 [0.78-0.86]	0.82 [0.77-0.87]	0.82 [0.77-0.88]	0.86 [0.81-0.90]	0.78 [0.72-0.83]
F	32	95	0.82 [0.76-0.87]	0.79 [0.73-0.86]	0.75 [0.62-0.87]	0.84 [0.78-0.90]	0.62 [0.48-0.74]	0.91 [0.86-0.96]
M	135	76	0.75 [0.70-0.80]	0.74 [0.69-0.80]	0.79 [0.72-0.84]	0.70 [0.61-0.78]	0.82 [0.76-0.88]	0.65 [0.56-0.73]
Qatar	109	78	0.82 [0.78-0.87]	0.81 [0.77-0.86]	0.86 [0.81-0.92]	0.77 [0.69-0.84]	0.84 [0.78-0.89]	0.80 [0.73-0.88]
US	58	93	0.72 [0.66-0.78]	0.70 [0.64-0.76]	0.62 [0.52-0.72]	0.78 [0.72-0.85]	0.64 [0.54-0.75]	0.77 [0.69-0.84]
**Aggregated raw classification CV metrics**								
ASDvsNon-ASD	167	171	0.79 [0.75-0.83]	0.79 [0.75-0.83]	0.80 [0.75-0.85]	0.78 [0.72-0.83]	0.78 [0.73-0.83]	0.80 [0.75-0.85]
ASDvsNT	167	128	0.83 [0.79-0.86]	0.83 [0.79-0.87]	0.80 [0.75-0.85]	0.86 [0.81-0.91]	0.88 [0.84-0.92]	0.77 [0.71-0.83]
F	32	95	0.83 [0.77-0.88]	0.81 [0.74-0.88]	0.78 [0.66-0.90]	0.84 [0.78-0.90]	0.62 [0.49-0.74]	0.92 [0.87-0.97]
M	135	76	0.77 [0.72-0.82]	0.76 [0.70-0.81]	0.81 [0.75-0.86]	0.70 [0.61-0.78]	0.83 [0.77-0.88]	0.67 [0.58-0.75]
Qatar	109	78	0.83 [0.79-0.88]	0.82 [0.78-0.87]	0.88 [0.83-0.93]	0.77 [0.69-0.84]	0.84 [0.79-0.89]	0.82 [0.75-0.90]
US	58	93	0.74 [0.68-0.79]	0.72 [0.66-0.78]	0.66 [0.55-0.75]	0.78 [0.72-0.85]	0.66 [0.56-0.76]	0.78 [0.71-0.85]
**Aggregated tuned classification hold-out metrics**								
ASDvsNon-ASD	61	59	0.72 [0.64-0.78]	0.72 [0.65-0.78]	0.62 [0.52-0.73]	0.81 [0.72-0.89]	0.78 [0.67-0.87]	0.68 [0.58-0.77]
ASDvsNT *	61	35	0.78 [0.71-0.85]	0.80 [0.74-0.87]	0.72 [0.62-0.81]	0.89 [0.79-0.97]	0.92 [0.85-0.98]	0.65 [0.53-0.76]
F	15	24	0.74 [0.62-0.85]	0.71 [0.59-0.84]	0.60 [0.38-0.82]	0.83 [0.70-0.95]	0.69 [0.45-0.91]	0.77 [0.62-0.90]
M	46	35	0.70 [0.62-0.79]	0.72 [0.64-0.79]	0.63 [0.51-0.74]	0.80 [0.68-0.90]	0.81 [0.69-0.91]	0.62 [0.50-0.74]
**Aggregated raw classification hold-out metric**								
ASDvsNon-ASD	61	59	0.72 [0.64-0.78]	0.72 [0.65-0.79]	0.66 [0.56-0.75]	0.78 [0.68-0.87]	0.75 [0.65-0.85]	0.69 [0.59-0.78]
ASDvsNT	61	35	0.75 [0.68-0.82]	0.79 [0.72-0.85]	0.66 [0.55-0.75]	0.91 [0.84-1.00]	0.93 [0.86-1.00]	0.60 [0.49-0.71]
F	15	24	0.74 [0.62-0.85]	0.71 [0.59-0.84]	0.60 [0.38-0.82]	0.83 [0.70-0.95]	0.69 [0.45-0.91]	0.77 [0.62-0.90]
M	46	35	0.70 [0.62-0.78]	0.71 [0.62-0.79]	0.67 [0.56-0.79]	0.74 [0.62-0.85]	0.78 [0.66-0.88]	0.63 [0.50-0.76]

Supplementary Table 7. Detailed aggregated raw and tuned classification CV and hold-out metrics of the Machine Learning Classifiers. M=male, F=female, ASDvsNon-ASD= whole dataset, ASDvsNT= dataset with the exclusion of the non-autism clinical subgroup. The decision threshold was calculated from the inner dataset aggregated across modules; one for the entire dataset, one with the exclusion of the non-autism clinical subgroup. The rows using the latter threshold are marked with an *.

Group	N_{ASD}	N_{nASD}	Accuracy [95% CI]	BA [95% CI]	Sensitivity [95% CI]	Specificity [95% CI]	PPV [95% CI]	NPV [95% CI]
**Few-to-no-words**								
ASDvsNon-ASD	112	33	0.80 [0.74-0.86]	0.72 [0.65-0.80]	0.87 [0.81-0.92]	0.58 [0.44-0.72]	0.87 [0.82-0.92]	0.56 [0.41-0.69]
ASDvsNT *	112	22	0.87 [0.81-0.91]	0.81 [0.73-0.89]	0.89 [0.84-0.94]	0.73 [0.56-0.88]	0.94 [0.90-0.98]	0.57 [0.41-0.72]
F	21	14	0.80 [0.69-0.91]	0.77 [0.65-0.89]	0.90 [0.80-1.00]	0.64 [0.42-0.87]	0.79 [0.65-0.93]	0.82 [0.60-1.00]
M	91	19	0.80 [0.74-0.85]	0.70 [0.60-0.79]	0.86 [0.79-0.91]	0.53 [0.33-0.71]	0.90 [0.84-0.95]	0.43 [0.25-0.61]
T	6	21	0.78 [0.63-0.89]	0.74 [0.56-0.92]	0.67 [0.33-1.00]	0.81 [0.67-0.95]	0.50 [0.20-0.80]	0.89 [0.76-1.00]
1	106	12	0.81 [0.75-0.86]	0.53 [0.43-0.62]	0.88 [0.83-0.93]	0.17 [0.00-0.38]	0.90 [0.85-0.95]	0.13 [0.00-0.33]
**Phrase speech**								
ASDvsNon-ASD	12	38	0.84 [0.76-0.92]	0.78 [0.66-0.90]	0.67 [0.43-0.89]	0.89 [0.81-0.97]	0.67 [0.43-0.90]	0.89 [0.80-0.97]
ASDvsNT *	12	21	0.85 [0.73-0.94]	0.82 [0.70-0.95]	0.75 [0.50-0.94]	0.90 [0.79-1.00]	0.82 [0.61-1.00]	0.86 [0.74-0.97]
F	1	19	0.85 [0.70-0.95]	0.45 [0.39-0.50]	0.00 [0.00-0.00]	0.89 [0.78-1.00]	0.00 [0.00-0.00]	0.94 [0.84-1.00]
M	11	19	0.83 [0.70-0.93]	0.81 [0.67-0.95]	0.73 [0.50-1.00]	0.89 [0.76-1.00]	0.80 [0.57-1.00]	0.85 [0.71-1.00]
**Fluent speech**								
ASDvsNASD	43	100	0.73 [0.67-0.80]	0.69 [0.62-0.76]	0.58 [0.45-0.70]	0.80 [0.73-0.87]	0.56 [0.43-0.67]	0.82 [0.75-0.88]
ASDvsNT *	43	85	0.77 [0.70-0.83]	0.73 [0.67-0.80]	0.65 [0.53-0.77]	0.82 [0.75-0.89]	0.65 [0.53-0.76]	0.82 [0.76-0.89]
F	10	62	0.82 [0.74-0.89]	0.69 [0.55-0.82]	0.50 [0.22-0.75]	0.87 [0.80-0.94]	0.38 [0.17-0.62]	0.92 [0.85-0.97]
M	33	38	0.65 [0.55-0.75]	0.65 [0.55-0.74]	0.61 [0.46-0.74]	0.68 [0.55-0.81]	0.62 [0.48-0.77]	0.67 [0.54-0.78]

Supplementary Table 8-a: Detailed tuned classification CV metrics of the Machine Learning Classifiers by language and development level and demographic subgroups. M=male, F=female, ASDvsNon-ASD= whole dataset, ASDvsNT= dataset with the exclusion of the non-autism clinical subgroup. The decision threshold was calculated from the inner dataset aggregated across modules; one for the entire dataset, one with the exclusion of the non-autism clinical subgroup. The rows using the latter threshold are marked with an *.

Group	N_{ASD}	N_{nASD}	Accuracy [95% CI]	BA [95% CI]	Sensitivity [95% CI]	Specificity [95% CI]	PPV [95% CI]	NPV [95% CI]
**Few-to-no-words**								
ASDvsNon-ASD	112	33	0.81 [0.77-0.87]	0.73 [0.66-0.80]	0.88 [0.83-0.93]	0.58 [0.44-0.72]	0.88 [0.83-0.93]	0.59 [0.45-0.73]
ASDvsNT	112	22	0.87 [0.83-0.92]	0.85 [0.78-0.92]	0.88 [0.83-0.93]	0.82 [0.67-0.95]	0.96 [0.93-0.99]	0.58 [0.43-0.72]
F	21	14	0.80 [0.69-0.91]	0.77 [0.65-0.89]	0.90 [0.80-1.00]	0.64 [0.42-0.87]	0.79 [0.65-0.93]	0.82 [0.60-1.00]
M	91	19	0.82 [0.75-0.87]	0.71 [0.61-0.80]	0.88 [0.82-0.93]	0.53 [0.33-0.71]	0.90 [0.85-0.95]	0.48 [0.28-0.65]
T	6	21	0.81 [0.70-0.93]	0.82 [0.68-0.96]	0.83 [0.50-1.00]	0.81 [0.67-0.95]	0.56 [0.29-0.83]	0.94 [0.84-1.00]
1	106	12	0.81 [0.75-0.87]	0.53 [0.43-0.63]	0.89 [0.84-0.93]	0.17 [0.00-0.38]	0.90 [0.86-0.95]	0.14 [0.00-0.33]
**Phrase speech**								
ASDvsNon-ASD	12	38	0.86 [0.78-0.94]	0.82 [0.71-0.93]	0.75 [0.54-0.94]	0.89 [0.81-0.97]	0.69 [0.47-0.92]	0.92 [0.83-0.98]
ASDvsNT	12	21	0.88 [0.79-0.97]	0.85 [0.73-0.97]	0.75 [0.50-0.94]	0.95 [0.86-1.00]	0.90 [0.73-1.00]	0.87 [0.74-0.97]
F	1	19	0.90 [0.80-1.00]	0.95 [0.69-1.00]	1.00 [0.00-1.00]	0.89 [0.78-1.00]	0.33 [0.00-1.00]	1.00 [1.00-1.00]
M	11	19	0.83 [0.70-0.93]	0.81 [0.67-0.95]	0.73 [0.50-1.00]	0.89 [0.76-1.00]	0.80 [0.57-1.00]	0.85 [0.71-1.00]
**Fluent speech**								
ASDvsNon-ASD	43	100	0.74 [0.68-0.80]	0.70 [0.63-0.77]	0.60 [0.48-0.72]	0.80 [0.73-0.87]	0.57 [0.44-0.68]	0.82 [0.76-0.88]
ASDvsNT	43	85	0.77 [0.70-0.83]	0.72 [0.65-0.80]	0.60 [0.47-0.72]	0.85 [0.78-0.91]	0.67 [0.54-0.78]	0.81 [0.74-0.88]
F	10	62	0.82 [0.74-0.89]	0.69 [0.55-0.82]	0.50 [0.22-0.75]	0.87 [0.80-0.94]	0.38 [0.17-0.62]	0.92 [0.85-0.97]
M	33	38	0.66 [0.56-0.76]	0.66 [0.56-0.76]	0.64 [0.49-0.77]	0.68 [0.55-0.81]	0.64 [0.50-0.78]	0.68 [0.56-0.80]

Supplementary Table 8-b: Detailed raw classification CV metrics of the Machine Learning Classifiers by language and development level and demographic subgroups. M=male, F=female, ASDvsNon-ASD= whole dataset, ASDvsNT= dataset with the exclusion of the non-autism clinical subgroup. The decision threshold was 0.5.

Group	N_{ASD}	N_{nASD}	Accuracy [95% CI]	BA [95% CI]	Sensitivity [95% CI]	Specificity [95% CI]	PPV [95% CI]	NPV [95% CI]
**Few-to-no-words**								
ASDvsNon-ASD	25	26	0.67 [0.57-0.78]	0.66 [0.55-0.77]	0.68 [0.52-0.83]	0.65 [0.50-0.81]	0.65 [0.50-0.82]	0.68 [0.52-0.83]
ASDvsNT *	25	12	0.84 [0.73-0.92]	0.86 [0.79-0.93]	0.76 [0.61-0.89]	1.00 [1.00-1.00]	1.00 [1.00-1.00]	0.67 [0.47-0.85]
F	7	10	0.71 [0.53-0.88]	0.70 [0.52-0.89]	0.71 [0.40-1.00]	0.70 [0.45-0.91]	0.62 [0.31-0.88]	0.78 [0.55-1.00]
M	18	16	0.65 [0.50-0.79]	0.64 [0.50-0.78]	0.67 [0.47-0.83]	0.62 [0.42-0.82]	0.67 [0.47-0.84]	0.62 [0.41-0.81]
T	8	10	0.72 [0.56-0.89]	0.69 [0.55-0.83]	0.38 [0.12-0.67]	1.00 [1.00-1.00]	1.00 [1.00-1.00]	0.67 [0.47-0.87]
1	17	16	0.64 [0.51-0.76]	0.61 [0.48-0.75]	0.82 [0.67-0.95]	0.44 [0.24-0.67]	0.61 [0.43-0.77]	0.70 [0.50-0.92]
**Phrase speech**								
ASDvsNon-ASD	20	18	0.71 [0.58-0.82]	0.71 [0.60-0.83]	0.60 [0.40-0.78]	0.83 [0.69-0.95]	0.80 [0.62-0.94]	0.65 [0.48-0.82]
ASDvsNT *	20	12	0.75 [0.62-0.88]	0.75 [0.61-0.89]	0.75 [0.58-0.90]	0.75 [0.54-1.00]	0.83 [0.67-1.00]	0.64 [0.42-0.85]
F	5	6	0.55 [0.27-0.82]	0.52 [0.34-0.69]	0.20 [0.00-0.50]	0.83 [0.50-1.00]	0.50 [0.00-1.00]	0.56 [0.27-0.83]
M	15	12	0.78 [0.63-0.89]	0.78 [0.65-0.91]	0.73 [0.53-0.93]	0.83 [0.64-1.00]	0.85 [0.67-1.00]	0.71 [0.50-0.92]
**Fluent speech**								
ASDvsNon-ASD	16	16	0.75 [0.62-0.88]	0.81 [0.71-0.91]	0.69 [0.47-0.87]	0.81 [0.64-0.94]	0.79 [0.60-0.93]	0.72 [0.53-0.88]
ASDvsNT *	16	12	0.75 [0.61-0.89]	0.80 [0.69-0.92]	0.75 [0.57-0.93]	0.75 [0.50-0.93]	0.80 [0.62-0.94]	0.69 [0.46-0.91]
F	3	8	1.00 [1.00-1.00]	1.00 [1.00-1.00]	1.00 [1.00-1.00]	1.00 [1.00-1.00]	1.00 [1.00-1.00]	1.00 [1.00-1.00]
M	13	8	0.62 [0.43-0.81]	0.77 [0.65-0.89]	0.62 [0.38-0.85]	0.62 [0.30-0.90]	0.73 [0.50-0.92]	0.50 [0.22-0.78]

Supplementary Table 9-a: Detailed tuned classification hold-out metrics of the Machine Learning Classifiers by language and development level and demographic subgroups. M=male, F=female, ASDvsNon-ASD= whole dataset, ASDvsNT= dataset with the exclusion of the non-autism clinical subgroup. The decision threshold was calculated from the inner dataset aggregated across modules; one for the entire dataset, one with the exclusion of the non-autism clinical subgroup. The rows using the latter threshold are marked with an *.

Group	N_{ASD}	N_{nASD}	Accuracy [95% CI]	BA [95% CI]	Sensitivity [95% CI]	Specificity [95% CI]	PPV [95% CI]	NPV [95% CI]
**Few-to-no-words**								
ASDvsNon-ASD	25	25	0.66 [0.54-0.76]	0.66 [0.55-0.77]	0.68 [0.52-0.82]	0.64 [0.47-0.79]	0.65 [0.48-0.80]	0.67 [0.50-0.83]
ASDvsNT	25	11	0.78 [0.67-0.89]	0.84 [0.76-0.92]	0.68 [0.50-0.83]	1.00 [1.00-1.00]	1.00 [1.00-1.00]	0.58 [0.38-0.76]
F	7	10	0.71 [0.53-0.88]	0.70 [0.52-0.89]	0.71 [0.40-1.00]	0.70 [0.45-0.91]	0.62 [0.31-0.88]	0.78 [0.55-1.00]
M	18	15	0.64 [0.48-0.76]	0.64 [0.50-0.77]	0.67 [0.47-0.84]	0.60 [0.38-0.80]	0.67 [0.47-0.85]	0.60 [0.38-0.80]
T	8	10	0.72 [0.56-0.89]	0.69 [0.55-0.83]	0.38 [0.12-0.67]	1.00 [1.00-1.00]	1.00 [1.00-1.00]	0.67 [0.47-0.87]
1	17	15	0.62 [0.47-0.75]	0.61 [0.48-0.74]	0.82 [0.67-0.95]	0.40 [0.19-0.62]	0.61 [0.43-0.77]	0.67 [0.38-0.90]
**Phrase speech**								
ASDvsNon-ASD	20	18	0.71 [0.58-0.82]	0.71 [0.60-0.83]	0.60 [0.40-0.78]	0.83 [0.69-0.95]	0.80 [0.62-0.94]	0.65 [0.48-0.82]
ASDvsNT	20	12	0.69 [0.56-0.81]	0.71 [0.59-0.84]	0.60 [0.42-0.76]	0.83 [0.64-1.00]	0.86 [0.69-1.00]	0.56 [0.37-0.75]
F	5	6	0.55 [0.27-0.82]	0.52 [0.34-0.69]	0.20 [0.00-0.50]	0.83 [0.50-1.00]	0.50 [0.00-1.00]	0.56 [0.27-0.83]
M	15	12	0.78 [0.63-0.89]	0.78 [0.65-0.91]	0.73 [0.53-0.93]	0.83 [0.64-1.00]	0.85 [0.67-1.00]	0.71 [0.50-0.92]
**Fluent speech**								
ASDvsNon-ASD	16	16	0.81 [0.69-0.91]	0.81 [0.71-0.92]	0.69 [0.47-0.87]	0.94 [0.82-1.00]	0.92 [0.77-1.00]	0.75 [0.57-0.89]
ASDvsNT	16	12	0.79 [0.64-0.89]	0.80 [0.69-0.92]	0.69 [0.50-0.88]	0.92 [0.77-1.00]	0.92 [0.75-1.00]	0.69 [0.50-0.88]
F	3	8	1.00 [1.00-1.00]	1.00 [1.00-1.00]	1.00 [1.00-1.00]	1.00 [1.00-1.00]	1.00 [1.00-1.00]	1.00 [1.00-1.00]
M	13	8	0.71 [0.52-0.86]	0.75 [0.61-0.89]	0.62 [0.38-0.85]	0.88 [0.67-1.00]	0.89 [0.70-1.00]	0.58 [0.31-0.82]

Supplementary Table 9-b: Detailed raw classification hold-out metrics of the Machine Learning Classifiers by language and development level and demographic subgroups. M=male, F=female, ASDvsNon-ASD= whole dataset, ASDvsNT= dataset with the exclusion of the non-autism clinical subgroup. The decision threshold was 0.5.

	Correctly diagnosed / sample number							
	Few-to-no-words group		Phrase speech group		Fluent speech group		Aggregated	
	Inner set	Hold-out set	Inner set	Hold-out set	Inner set	Hold-out set	Inner set	Hold-out set
NT	18/22	11/11	20/21	10/12	72/85	12/12	110/128	33/35
non-ASD Clinical	1/11	6/14	14/17	5/6	8/15	4/4	23/43	15/24
ASD	97/112	16/25	8/12	12/20	25/43	10/16	130/167	38/61

Supplementary Table 10: Detailed classification CV and hold-out metrics of the Machine Learning Classifiers by ADOS-2 Module and diagnostic subgroups. Subjects having multiple non-autism clinical diagnoses are included in all corresponding rows of the table.

	MWW H	MWW p-value	MWW power
**Few-to-no-words group**	35.36	2.74E-9	0.84
**Phrase speech group**	15.99	6.38E-5	0.88
**Fluent speech group**	29.37	5.98E-8	0.78

Supplementary Table 11: Mann-Whitney-Wilcoxon rank sum test of the ML outputs. To explore distributional differences in the model confidence, predicted probability estimates were grouped by ground-truth class (ASD vs. Non-ASD), and a Mann-Whitney-Wilcoxon rank sum test was conducted (7), to assess whether the distributions differ significantly at the given sample sizes. A similar method was used in (8) to differentiate between probability outputs of machine learning models in case of valid and invalid test-cases.

	N_{ASD}	N_{nASD}	**Accuracy**	**Sensitivity**	**Specificity**
**ADHD**	57	17	0.78	0.84	0.59
**ID/DD**	12	15	0.56	0.67	0.47
**FXS**	17	12	0.76	0.94	0.50

Supplementary Table 12. Classification metrics for the main comorbidities from the whole dataset (training set and hold-out test set). The table showcases all available cohorts (Few-to-no-words-, Phrase speech- and Fluent speech group).

**Dataset**	**Site**	**Group**	N_{ASD}	N_{nASD} / N_{NT}	**Accuracy [95% CI]**	**Sensitivity [95% CI]**	**Specificity [95% CI]**	**PPV [95% CI]**	**NPV [95% CI]**
**Inner set**	**US**	**ASD - non-ASD**	58	93	0.72 [0.66-0.78]	0.62 [0.52-0.72]	0.78 [0.72-0.85]	0.64 [0.54-0.75]	0.77 [0.69-0.84]
		**ASD-NT**	58	80	0.73 [0.67-0.80]	0.69 [0.59-0.79]	0.76 [0.68-0.84]	0.68 [0.58-0.78]	0.77 [0.70-0.85]
	**Qatar**	**ASD - non-ASD**	109	78	0.82 [0.78-0.87]	0.86 [0.81-0.92]	0.77 [0.69-0.84]	0.84 [0.78-0.89]	0.80 [0.73-0.88]
		**ASD-NT**	109	48	0.90 [0.86-0.94]	0.89 [0.84-0.94]	0.92 [0.85-0.98]	0.96 [0.93-0.99]	0.79 [0.69-0.87]
**Hold-out set**	**US**	**ASD - non-ASD**	31	29	0.72 [0.62-0.82]	0.68 [0.54-0.81]	0.76 [0.62-0.88]	0.75 [0.62-0.88]	0.69 [0.56-0.82]
		**ASD-NT**	31	19	0.82 [0.72-0.90]	0.77 [0.62-0.89]	0.89 [0.75-1.00]	0.92 [0.83-1.00]	0.71 [0.55-0.86]
	**Qatar**	**ASD - non-ASD**	30	30	0.72 [0.62-0.80]	0.57 [0.42-0.70]	0.87 [0.76-0.96]	0.81 [0.65-0.95]	0.67 [0.55-0.78]
		**ASD-NT**	30	16	0.74 [0.63-0.85]	0.67 [0.53-0.81]	0.88 [0.73-1.00]	0.91 [0.80-1.00]	0.58 [0.41-0.75]

Supplementary Table 13. Detailed aggregated raw and tuned classification CV and hold-out metrics of the Machine Learning Classifiers by the country of origin (US and Qatar). ASDv - non-ASD= whole dataset, ASD-NT= dataset with the exclusion of the non-autism clinical

subgroup. The decision threshold was calculated from the inner dataset aggregated across modules; one for the entire dataset, one with the exclusion of the non-autism clinical subgroup.

		CV metrics					Hold-out metrics				
	**Model**	**N_{ASD}**	**N_{nASD}**	**Accuracy**	**Sensitivity**	**Specificity**	**N_{ASD}**	**N_{nASD}**	**Accuracy**	**Sensitivity**	**Specificity**
**Few-to-no-words**	Joint	112	33	0.83	*0.96*	*0.42*	25	25	0.56	*0.92*	*0.20*
	Separate			0.81	**0.88**	**0.58**			0.66	**0.68**	**0.64**
**Phrase speech**	Joint	12	38	0.80	0.75	0.82	20	18	0.66	0.55	0.78
	Separate			0.86	0.75	0.89			0.71	0.60	0.83
**Fluent speech**	Joint	42	100	0.76	*0.40*	*0.91*	16	16	0.72	*0.44*	*1.00*
	Separate			0.74	**0.60**	**0.80**			0.81	**0.69**	**0.94**
**Aggregated**	Joint	166	171	0.80	0.80	0.80	61	59	0.63	0.67	0.59
	Separate			0.79	0.80	0.78			0.72	0.66	0.78

Supplementary Table 14: Justification of the model stratification by language and developmental level. The table lists raw classification CV and hold-out metrics (decision threshold at 0.5) of the Machine Learning Classifiers by language and development level for ASD vs. nASD cohorts. The "Joint" and "Separate" entries compare models trained on subjects from all and only distinct developmental levels (for the joint model the developmental level is added as a categorical input feature). While the aggregated CV metrics do not show difference between the two methods, the hold-out metrics do show some improvement for the separate models. Even more importantly, the module-wise results show a deeper, structural difference. The values in bold show where the joint model was able to learn differences between the behavioral patterns of younger non-ASD and older ASD subjects. The values in italic show where the joint model was unable to learn differences between behavioural patterns of younger non-ASD and older ASD subjects, showing high sensitivity - poor specificity in the younger, and low sensitivity - high specificity in the older developmental levels.

3. References

1. Koluguri NR, Park T, Ginsburg B. TitaNet: Neural Model for speaker representation with 1D Depth-wise separable convolutions and global context [Internet]. arXiv; 2021 [cited 2025 Oct 31]. Available from: https://arxiv.org/abs/2110.04410 doi:10.48550/ARXIV.2110.04410

2. Tafasca S, Gupta A, Kojovic N, Gelsomini M, Maillart T, Papandrea M, et al. The AI4Autism Project: A Multimodal and Interdisciplinary Approach to Autism Diagnosis and Stratification. In: International Conference on Multimodal Interaction [Internet]. Paris France: ACM; 2023 [cited 2025 Sep 25]. p. 414–25. Available from: https://dl.acm.org/doi/10.1145/3610661.3616239

doi:10.1145/3610661.3616239

3. Lau JCY, Patel S, Kang X, Nayar K, Martin GE, Choy J, et al. Cross-linguistic patterns of speech prosodic differences in autism: A machine learning study. Pegoraro C, editor. PLOS ONE. 2022 Jun 8;17(6):e0269637. doi:10.1371/journal.pone.0269637

4. Stuart N, Whitehouse A, Palermo R, Bothe E, Badcock N. Eye Gaze in Autism Spectrum Disorder: A Review of Neural Evidence for the Eye Avoidance Hypothesis. J Autism Dev Disord. 2023 May;53(5):1884–905. doi:10.1007/s10803-022-05443-z

5. Begeer S, Koot HM, Rieffe C, Meerum Terwogt M, Stegge H. Emotional competence in children with autism: Diagnostic criteria and empirical evidence. Dev Rev. 2008 Sep;28(3):342–69. doi:10.1016/j.dr.2007.09.001

6. Trevisan DA, Hoskyn M, Birmingham E. Facial Expression Production in Autism: A Meta‐Analysis. Autism Res. 2018 Dec;11(12):1586–601. doi:10.1002/aur.2037

7. MacFarland TW, Yates JM. Mann–Whitney U Test. In: Introduction to Nonparametric Statistics for the Biological Sciences Using R [Internet]. Cham: Springer International Publishing; 2016 [cited 2025 Jun 30]. p. 103–32. Available from: http://link.springer.com/10.1007/978-3-319-30634-6_4 doi:10.1007/978-3-319-30634-6_4

8. Taherkhani H, Hemmati H. VALTEST: Automated Validation of Language Model Generated Test Cases. ArXiv Prepr ArXiv241108254. 2024.
